# Clinical heterogeneity of Pulmonary Arterial Hypertension associated with variants in *TBX4*

**DOI:** 10.1371/journal.pone.0232216

**Published:** 2020-04-29

**Authors:** Ignacio Hernandez-Gonzalez, Jair Tenorio, Julian Palomino-Doza, Amaya Martinez Meñaca, Rafael Morales Ruiz, Mauro Lago-Docampo, María Valverde Gomez, Javier Gomez Roman, Ana Belén Enguita Valls, Carmen Perez-Olivares, Diana Valverde, Joan Gil Carbonell, Elvira Garrido-Lestache Rodríguez-Monte, Maria Jesus del Cerro, Pablo Lapunzina, Pilar Escribano-Subias

**Affiliations:** 1 Department of Cardiology, Hospital Universitario Río Hortega, Valladolid, Spain; 2 Institute of Medical and Molecular Genetics (INGEMM), Hospital Universitario La Paz, IdiPAZ, Universidad Autónoma de Madrid, Madrid, Spain; 3 Centro de Investigación Biomédica en Red de Enfermedades Raras (CIBERER), Institto de Salud Carlos III, Madrid, Spain; 4 Department of Cardiology, Inherited Cardiac Disease Unit, Hospital Universitario Doce de Octubre, Madrid, Spain; 5 Department of Cardiology, Pulmonary Hypertension Unit, Hospital Universitario Doce de Octubre, Madrid, Spain; 6 Centro de Investigación Biomedica en Red en Enfermedades Cardiovasculares, Institto de Salud Carlos III (CIBERCV), Madrid, Spain; 7 Department of Pneumology, Pulmonary Hypertension Unit, Lung Transplant Unit, Hospital Universitario Marqués de Valdecilla, Santander, Spain; 8 Department of Radiology, Pulmonary Hypertension Unit, Hospital Universitario Doce de Octubre, Madrid, Spain; 9 Department of Biochemistry, Genetics and Immunology, University of Vigo, Vigo, Spain; 10 Department of Pathology, Hospital Universitario Marqués de Valdecilla, Santander, Spain; 11 Department of Pathology, Pulmonary Hypertension Unit, Hospital Universitario Doce de Octubre, Madrid, Spain; 12 Department of Pneumology, Hospital de Alicante, Alicante, Spain; 13 Paediatric Cardiology and Grown Up Congenital Heart Disease Department, Hospital Universitario Ramón y Cajal, Madrid, Spain; Vanderbilt University Medical Center, UNITED STATES

## Abstract

**Background:**

The knowledge of hereditary predisposition has changed our understanding of Pulmonary Arterial Hypertension. Genetic testing has been widely extended and the application of Pulmonary Arterial Hypertension specific gene panels has allowed its inclusion in the diagnostic workup and increase the diagnostic ratio compared to the traditional sequencing techniques. This is particularly important in the differential diagnosis between Pulmonary Arterial Hypertension and Pulmonary Venoocclusive Disease.

**Methods:**

Since November 2011, genetic testing is offered to all patients with idiopathic, hereditable and associated forms of Pulmonary Arterial Hypertension or Pulmonary Venoocclusive Disease included in the Spanish Registry of Pulmonary Arterial Hypertension. Herein, we present the clinical phenotype and prognosis of all Pulmonary Arterial Hypertension patients with disease-associated variants in *TBX4*.

**Results:**

Out of 579 adults and 45 children, we found in eight patients from seven families, disease-causing associated variants in *TBX4*. All adult patients had a moderate-severe reduction in diffusion capacity. However, we observed a wide spectrum of clinical presentations, including Pulmonary Venoocclusive Disease suspicion, interstitial lung disease, pulmonary vascular abnormalities and congenital heart disease.

**Conclusions:**

Genetic testing is now essential for a correct diagnosis work-up in Pulmonary Arterial Hypertension. *TBX4*-associated Pulmonary Arterial Hypertension has marked clinical heterogeneity. In this regard, a genetic study is extremely useful to obtain an accurate diagnosis and provide appropriate management.

## 1. Introduction

Pulmonary Arterial Hypertension (PAH) is a severe and uncommon disease affecting small pulmonary arteries with an increase in pulmonary vascular resistance, which leads to right ventricle failure and death [1]. In the last two decades, knowledge of hereditary predisposition to PAH has drastically increased. In 2000, mutations in the Bone Morphogenic Protein Receptor Type 2 (BMPR2) gene were identified in families with heritable PAH [2, 3]. Since then, the importance of genetic testing and counselling has been steadily increasing. Genetic testing has been included in the diagnostic workup in the European Respiratory Society (ERS) and the European Society of Cardiology guidelines (ESC) [4].

The initial molecular approach to PAH testing was the use of Sanger sequencing. However, this is no longer appropriate due to a large number of genes currently known to be associated with the condition. To date, 12 genes have been associated with PAH with a high level of evidence and 5 with a low level of evidence [5]. Furthermore, the hypothesis of a digenic pattern of inheritance makes necessary novel approaches using Next Generation Sequencing (NGS) to obtain information from a set of genes, the entire exome or even the whole genome [6].

One of these recently associated genes encodes the T-box transcription factor 4 (*TBX4*), which is involved in the regulation of embryonic developmental processes. Pathogenic variants in *TBX4* are a well-established cause of PAH in children, typically associated with skeletal disorders (small patella syndrome, MIM #147891), intellectual disabilities and other cardiovascular disorders [7–11]. Mutations in this gene might also lead to diffuse developmental disorders in the lungs in neonates [12, 13].

Pulmonary Venooclusive Disease (PVOD) is the most lethal subtype of PAH, with different clinical, histological and genetic features [14–16]. Definitive diagnosis can be established by either histological analysis or by the presence of biallelic pathogenic variants in the Eukaryotic Translation Initiation Factor 2 Alpha Kinase 4 (*EIF2AK4*) gene [4]. Clinical diagnosis includes the combination of very low diffusion capacity (DLCO), resting hypoxemia, severe desaturation on exercise and a characteristic radiological pattern (ground-glass opacification, interlobular septal thickening and mediastinal lymphadenopathy) [14]. However, the differential diagnosis between PAH and PVOD represents a clinical challenge and misdiagnosis is a common problem [17, 18]. The importance of an accurate diagnosis lies in the specific management that must be provided to PVOD patients: close follow-up and early referral to lung transplantation units [4]. When PVOD is suspected, pulmonary vasodilators should be prescribed with caution due to the risk of pulmonary oedema and respiratory insufficiency worsening [14, 15].

PH associated with Congenital Heart Disease (CHD) is a PAH subtype and represents a heterogeneous group of patients. Patients with CHD have an increased pulmonary blood flow that can lead to unfavourable vascular remodelling and PH. It is estimated that 5–10% of patients with CHD develop PH [19]. However, the role of genetic mutations in the development of pulmonary vascular disease remains unclear.

This study aimed to describe the phenotype of PAH associated with *TBX4* variants. Herein, we present data from Spain’s nationwide PAH registry to identify clinical, radiological and histological patterns of *TBX4* variants.

## 2. Methods

### Study patients

Patients with idiopathic, hereditable and associated forms of PAH, and PVOD included the Spanish Registry of Pulmonary Arterial Hypertension (REHAP) and the Registry of Pediatric Pulmonary Hypertension Patients (REHIPED) were eligible for this study. REHAP and REHIPED are observational national registries with researching purposes that include patients with Group 1 and Group 4 Pulmonary Hypertension. A full list of REHAP and REHIPED centers and investigators is provided in the Supporting Information.

Pulmonary Arterial Hypertension was defined according to the 2015 ERS/ESC Guidelines for the Diagnosis and Treatment of Pulmonary Hypertension [4]. Right Heart Catheterism (RHC) at diagnosis includes Right Atrium Pressure, Mean Pulmonary Artery Pressure, Pulmonary Wedge Pressure, Cardiac Output, Cardiac Index and Pulmonary Vascular Resistance. Pulmonary vasoreactivity testing was performed in Idiopathic PAH (IPAH), Hereditable PAH (HPAH) and drug-induced PAH. Routine diagnostic workup included medical history, physical examination, 6-minute Walking Test (6MWT), echocardiogram, Multidetector Computed Tomography (MDCT), ventilation/perfusion lung scan, pulmonary function tests (PFT), and screening of Connective Tissue Disease, HIV infection and Portal Hypertension. PFT included the diffusing capacity for carbon monoxide (DLCO), which was considered moderately reduced when DLCO 43–62% of predicted values and severely reduced when DLCO < 43% of predicted values [20]. For this study, a comprehensive assessment was conducted to identify other conditions typically associated with TBX4 variants: skeletal disorders, congenital heart disease, intellectual impairment, or neurological disorders. Therapeutic management is left to the discretion of individual physicians.

Since November 2011, genetic studies have been offered to all patients included in REHAP and REHIPED registries with idiopathic, hereditable and associated forms of PAH, and PVOD. The ethical principles of the European Board of Medical Genetics and the 2015 ERS/ESC Guidelines for the Diagnosis and Treatment of Pulmonary Hypertension are to offer accurate information on the range of options available to make informed decisions and to allow equal access to genetic counselling and testing [4]. Pre and post-test genetic counselling were provided. All patients or legal tutors included in the analysis gave their written informed consent and the project was approved by the ethical committee for scientific research of the participant centers. We obtained written parental consent from parents or guardians of minors included in this study. A full list of REHAP and REHIPED centers and investigators is provided in the Supporting Information. In the pre-test visit, family history information was collected, but only probands were studied. When a positive result was observed, a genetic study was offered to first degree relatives when available. Cascade or cosegregation genetic tests were also performed. When an unaffected carrier was identified, a complete diagnostic was performed, including electrocardiogram, echocardiogram, N-terminal pro-brain natriuretic peptide (NT-proBNP) and 6 Minute Walking Test. Due to the typical phenotype, DLCO was also determined in healthy carriers. This evaluation is periodically repeated. When a sustained suspicion of early-stage PAH was observed, RHC was performed to rule out the condition.

### Molecular analysis

A PAH-specific 21 genes NGS panel was designed (HAP v1.2). Genes included are divided into: TGF-β signalling pathway-related genes (*BMPR2*, *BMPR1B*, *GDF2*, *SMAD1*, *SMAD4*, *SMAD5*, *SMAD9*, *ENG*, *ACVRL1* and *CAV1*); recently described genes in PAH (*KCNK3*, *KCNA5*, *NOTCH3*, *TBX4*, *TOPBP1*, *MMACHC9*); genes associated with PVOD (*EIF2AK4*) and candidate genes (*SARS2*, *CPS1*, *ABCC8*, *CBLN2*).

HAP v1.2 was designed with NimbleDesign (Roche, EEUU). Fragmentation and capture of the target regions were performed with SeqCap EZ Choice Enrichment Kit (Roche, EEUU) and sequencing was carried out in the IlluminaMiSeq platform (Illumina, EEUU). The in-house bioinformatics pipeline was developed to analyze the raw data. After the filtering of the relevant variants, validation of the candidate variants was carried out through traditional Sanger Sequencing. Review, classification and interpretation of the variants were made according to the American College of Medical Genetics and Genomics (ACMG) guidelines [21].

Copy Number Variations (CNV) analysis was performed by applying a custom script named “LACONv” (https://github.com/kibanez/LACONv), which has been developed in-house. It detects gains and losses in the genes included in the HAP v1.2 panel. Minimal depth by sample to be considered: 20X; Minimal depth in genomic intervals to be considered: 15X; Doses rate threshold for deletions: 0.60; Doses rate threshold for duplications: 1.20; Zscore threshold to establish CNVs (deletions): -2.000. Single Nucleotide Polymorphism (SNP) microarray validation of detected deletion through NGS was performed (Infinium OmniExpressExome-8 v1.6 Kit Illumina).

Pathogenicity prediction has been calculated by nine tools included in the dbNSFP algorithm plus CADD. Values to classify according to each predictor is detailed in Liu et al., 2015, Hum Mut (hay que meter la referencia, PMID: 26555599). For CADD, we established a threshold of 14 of the Phred score, being those values above it classified as damaging and the rest as neutral.

### Radiological assessment

Multidetector Computed Tomography (MDCT) images, when available, were reviewed by a cardiothoracic radiologist expert in PH imaging. Images were assessed for the presence of ground-glass opacification, interlobular septal lines, lymph node enlargement, pleural effusion, pericardial effusion and other remarkable findings. When two or more radiological abnormalities typical of PVOD (grounds grass opacification, interlobular septal thickening or mediastinal lymphadenopathy) were present, the radiological diagnosis was possible PVOD [22].

### Histological analysis

Histopathological evaluation was performed using hematoxylin-eosin-stained 5 μm slides from formalin-fixed paraffin-embedded lung obtained during a biopsy or after lung transplantation. Representative sections from each lobe of both lungs were obtained after lung transplantation. Specimens included vascular and bronchial surgical margin, hilar lymph nodes, and five or more representative samples of each lobe. Any remarkable macroscopic finding was included. Pulmonary arteries were evaluated for PAH signs such as medial hypertrophy/hyperplasia, intimal and adventitial fibrosis, and thrombotic lesions, plexiform lesions [23]. PVOD involvement was defined as an extensive and diffuse obstruction of pulmonary veins and venules by intimal thickening, with either fibrosis, cellular proliferation, or muscularization [14, 23]. Samples were also evaluated for the presence of parenchymal lung disease, diffuse developmental disorders, and growth abnormalities [24].

## 3. Results

579 adults and 45 children have been included in this study. Baseline characteristics of the adult and the pediatric cohorts are shown in Tables 1 and 2. Six different heterozygous variants were identified in the 8 subjects and consisted of 2 Copy Number Variants involving the 17q23.2 locus and 4 TBX4 Single Nucleotide Variants. Two of these mutations had been previously reported by our group and by Galambos et al., respectively [8, 15]. See Table 3 for a summary of patients and Table 4 for variants analysis. Furthermore, two Variants of Unknown Significance were identified in two adult patients with children-onset PAH. Clinical phenotypes, available histological analysis (patients 1 and 3) and variants analysis of each family are provided below.

**Table 1:** Baseline characteristics of adult cohort.

Age, years	45 (± 0,76)
Female sex	411 (71%)
**Etiology**	
Idiopathic	262 (45.25%)
Familial	31 (5.35%)
Pulmonary Venooclusive Disease	54 (9.33%)
Connective Tissue Disease	91 (15.72%)
Congenital Heart Disease	88 (15.2%)
Drugs	22 (3.8%)
Portopulmonary Hypertension	11 (1.9%)
Hereditary Hemorrhagic Telangiectasia	7 (1.21%)
Human Immunodeficiency Virus	13 (2.25%)
**Race**	
White	510 (88.1%)
Hispanic	37 (6.4%)
Romani	23 (4%)
Black	1 (0.2%)
North African	6 (1%)
Asian	1 (0.2%)
Hindu	1 (0.2%)

Data are median (range), mean (SD), or n (%).

**Table 2:** Baseline characteristics of paediatric cohort.

Age, years	9.9 (± 4.4)
Female sex	27 (60%)
**Etiology**	
Idiopathic	23 (51.1%)
Familial	4 (8.9%)
Pulmonary Venooclusive Disease	3 (6.7%)
Congenital Heart Disease	11 (24.4%)
Other	4 (8.9%)
**Race**	
White	39 (86.7%)
Hispanic	3 (6.7%)
Romani	3 (6.7%)

Data are median (range), mean (SD), or n (%).

**Table 3:** Clinical characteristics.

	Patient 1	Patient 2	Patient 3	Patient 4	Patient 5	Patient 6	Patient 7	Patient 8
**PAH-onset**	Adult	Adult	Adult	Adult	Adult	Child	Child	Child
**Initial Diagnosis**	Familial	Familial	Idiopathic	Idiopathic	Idiopathic	CHD	Idiopathic	Idiopathic
**Gender**	Female	Female	Female	Male	Male	Male	Female	Female
**Follow-up, years**	11	4	9	2.1	5	16	8	1.5
**Final status**	Alive	Alive	Transplant	Death	Alive	Alive	Alive	Alive
**Radiological Diagnosis**	ILD	PAH	PVOD	PVOD	PAH	PAH	PAH	ILD
**Skeletal Disorder**	Absent	Absent	Absent	Absent	Absent	Arthritis	Growth delay	Absent
**CHD**	Absent	Absent	Absent	Absent	Absent	OS ASD	Absent	PDA, PFO
**Neurologic/ psychomotor deficits**	Absent	Absent	Absent	Absent	Absent	Absent	Developmental delay Nystagmus	Hearing loss
**DLCO, % predicted**	34	29	55	32	61	49	NA	NA
**VFC, % predicted**	56	98	86	115	58	70	NA	NA
**FEV1, % predicted**	53	82	66	104	140	70	NA	NA
**mPAP, mmHg**	50	49	75	38	49	61	36	39
**PCWP, mmHg**	8	7	24	9	7	10	12	11
**CO, l/min**	3.8	2.8	3.5	3.45	6.3	3.1	2.9	1.6
**CI, l/min/m**^**2**^	2.3	1.99	2.02	1.95	3.4	1.5	4.9	4.7
**PVR, WU**	11	15	15	8.4	6.7	16.5	4.9*	6*
**AVT**	Negative	Negative	Negative	Negative	Positive	NA	Positive	Positive
**Treatment**	ERA + PDE5i + syst PC	ERA + PDE5i + Syst PC	ERA + PDE5i + syst PC	ERA + PDE5i	CCB + ERA	ERA + PDE5i + syst PC	ERA + PDE5i + CCB	PDE5i + syst PC

DLCO: Diffusing Capacity of the Lung for Carbon Monoxide; VFC: Vital Forced Capacity; FE1V: Forced Expiratory Volume in 1 second; mPAP: mean Pulmonary Artery Pressure; PCWP: Pulmonary Capillary Wedge Pressure; CO: Cardiac Output; CI: Cardiac Index; PVR: Pulmonary Vascular Resistance; WU: Wood Units; AVT: Acute Vasoreactivity Testing; ERA: Endothelin Receptor Antagonist; PDE-5i: Phosphodiesterase Type 5 Inhibito; CCB: Calcium Channel Blocker; PAH: Pulmonary Artery Hypertension; PVOD: Pulmonary Venooclusive Disease; CHD: Congenital Heart Disease; Syst PC: systemic prostacyclin; PDA: Patent Ductus Arteriosus; PFO: Patent Foramen Ovale; ILD: Interstitial Lung Disease

* Indexed PVR, UW.m2

**Table 4:** Variants analysis.

Family	Genomic coordinate (hg19)	cDNA and protein location	Exon/ Intron	Mutation type	Population frequency^†^	Pathogenicity predictors ^‡^	ACMG prediction ^§^	Inheritance^¶^	Reference
1	chr17:59543206dupAAG	c.308_310dupAAG: p.(Lys103_Val104insGlu)	3	duplication	0	1/1	P	*maternal*	Navas et al [25]
2	chr17:59544901G>T	c.432G>T:p.(Met144Ile)	4	missense	4.06e^-06^	8/10	LP	*maternal*	This study
3	chr17:59544901G>T	c.432G>T:p. (Met144Ile)	4	missense	4.06e^-06^	8/10	LP	*PD*	This study
4	chr17:59557681G>A	c.1021+1G>A	7	splicing	0	4/4	P	N/D	This study
5	chr17:59544901G>T	c.1019C>T:p. (Arg340*)	8	nonsense	0	8/10	P	*N/D*	Galambos et al. [8]
6	TBX4 deletion	N/A	N/A	N/A	N/A	N/A	LP	*De novo*	Kerstjens-Frederikse et al [7]
7	TBX4 deletion	N/A	N/A	N/A	N/A	N/A	LP	*De novo*	Kerstjens-Frederikse et al [7]

^†^ gnomAD exomes, gnomAD genomes, Kaviar, 1000G phase III, ESP

^‡^ dbNSFP (MutationTaster, MutationAssessor, FATHMM, FATHMM-MKL, MetaSVM, MetalR, Provean, LRT, SIFT)

^§^ ACMG prediction: P:Pathogenic, LP: Likely Pathogenic

^¶^ N/R: Not done; PD: parents death

### Family 1

**Fig 1:**
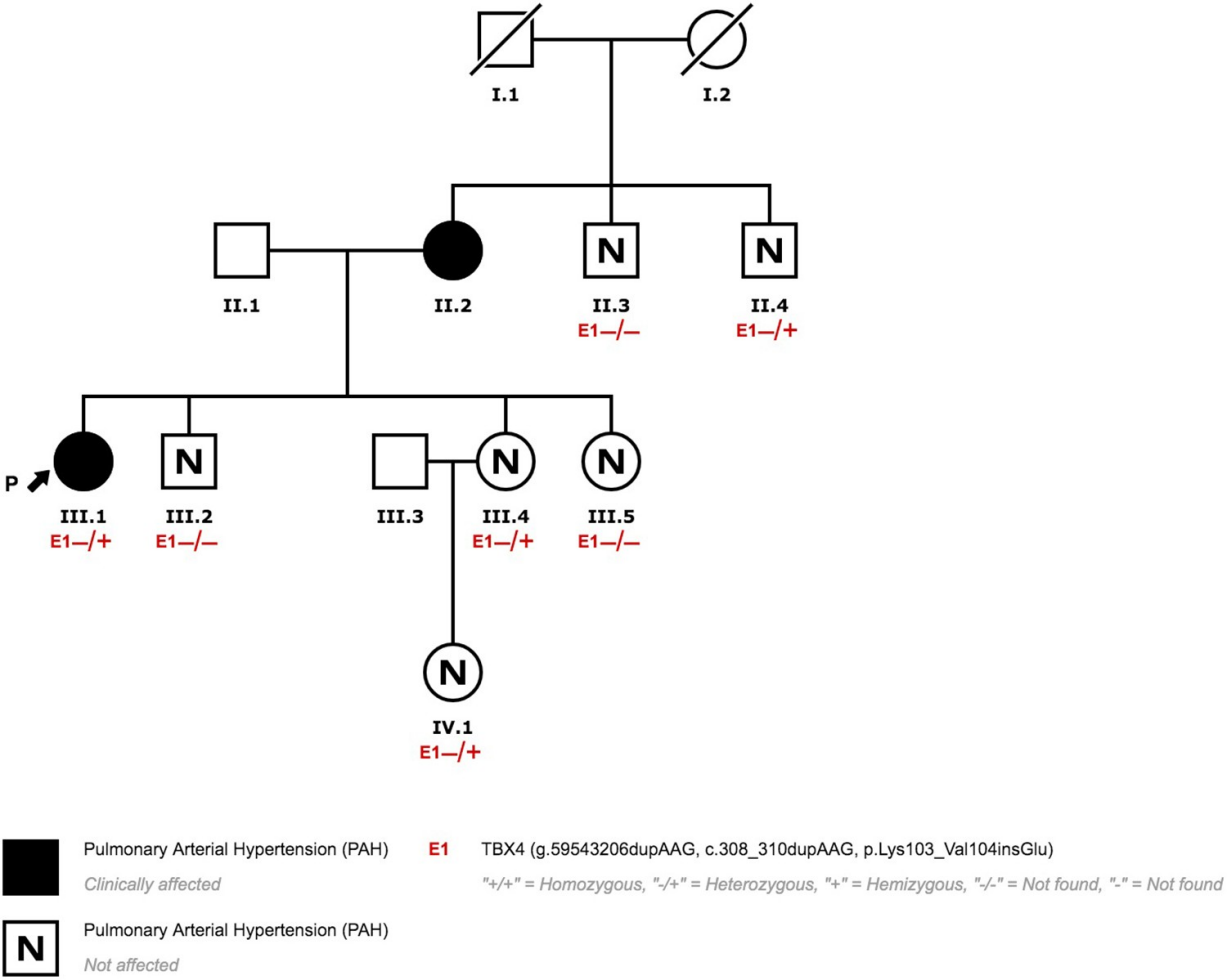
Family 1 pedigree.

Patient 1 and patient 2 (patient 1 mother) presented a pathogenic variant in *TBX4* (NM_018488.3:c.308_310dupAAG;p.Lys103_Val104insGlu) previously published by our group (Fig 1) [25]. Segregation analysis revealed that three other family members also carry the variant (patient 1 uncle, patient 1 sister, patient 1 niece). PAH was initially ruled out in relatives and carriers are periodically evaluated.

**Fig 2:**
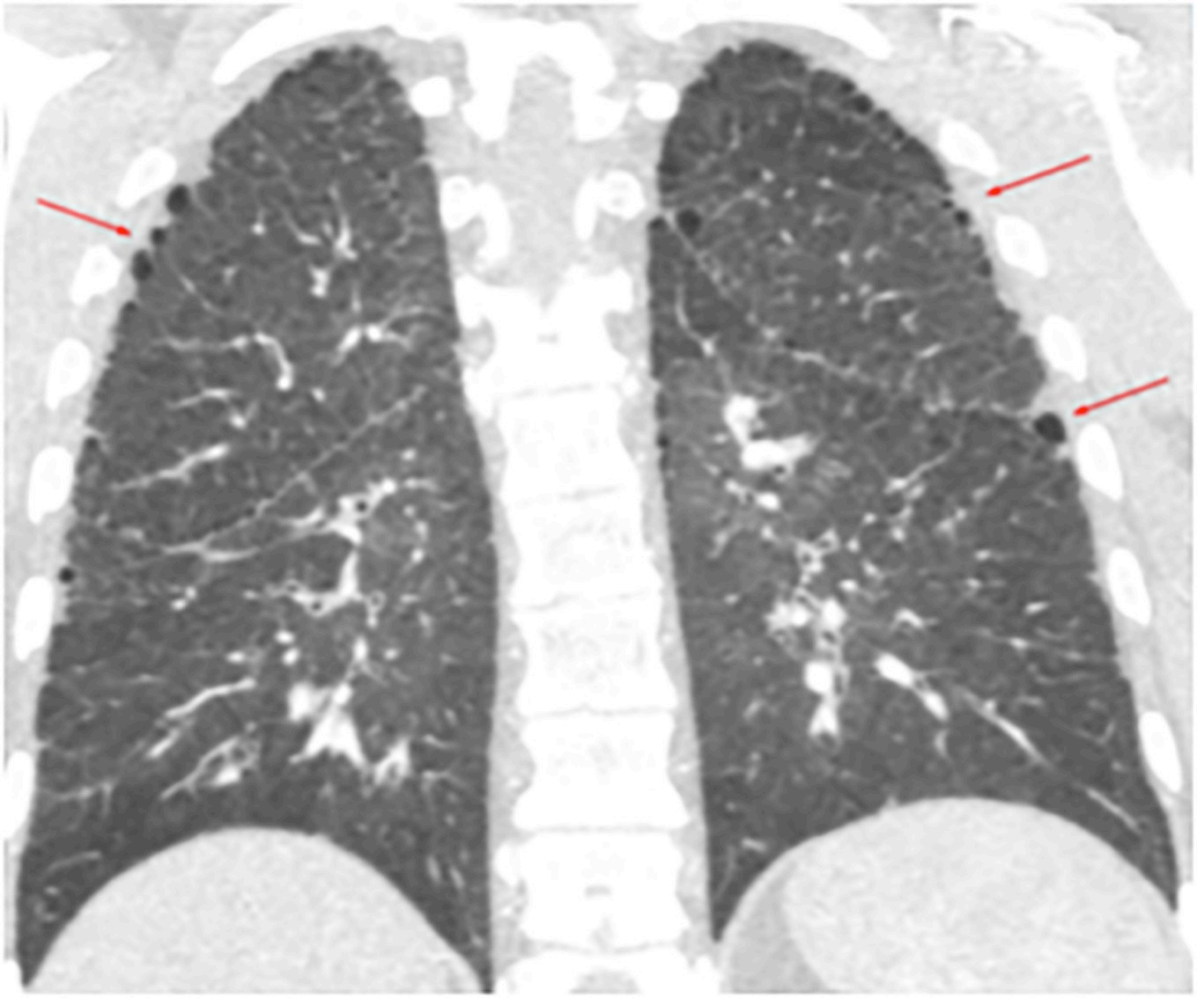
Patient 1 Multidetector Computed Tomography. Subtle emphysema radiological pattern with peripheral distribution (red arrows).

**Fig 3:**
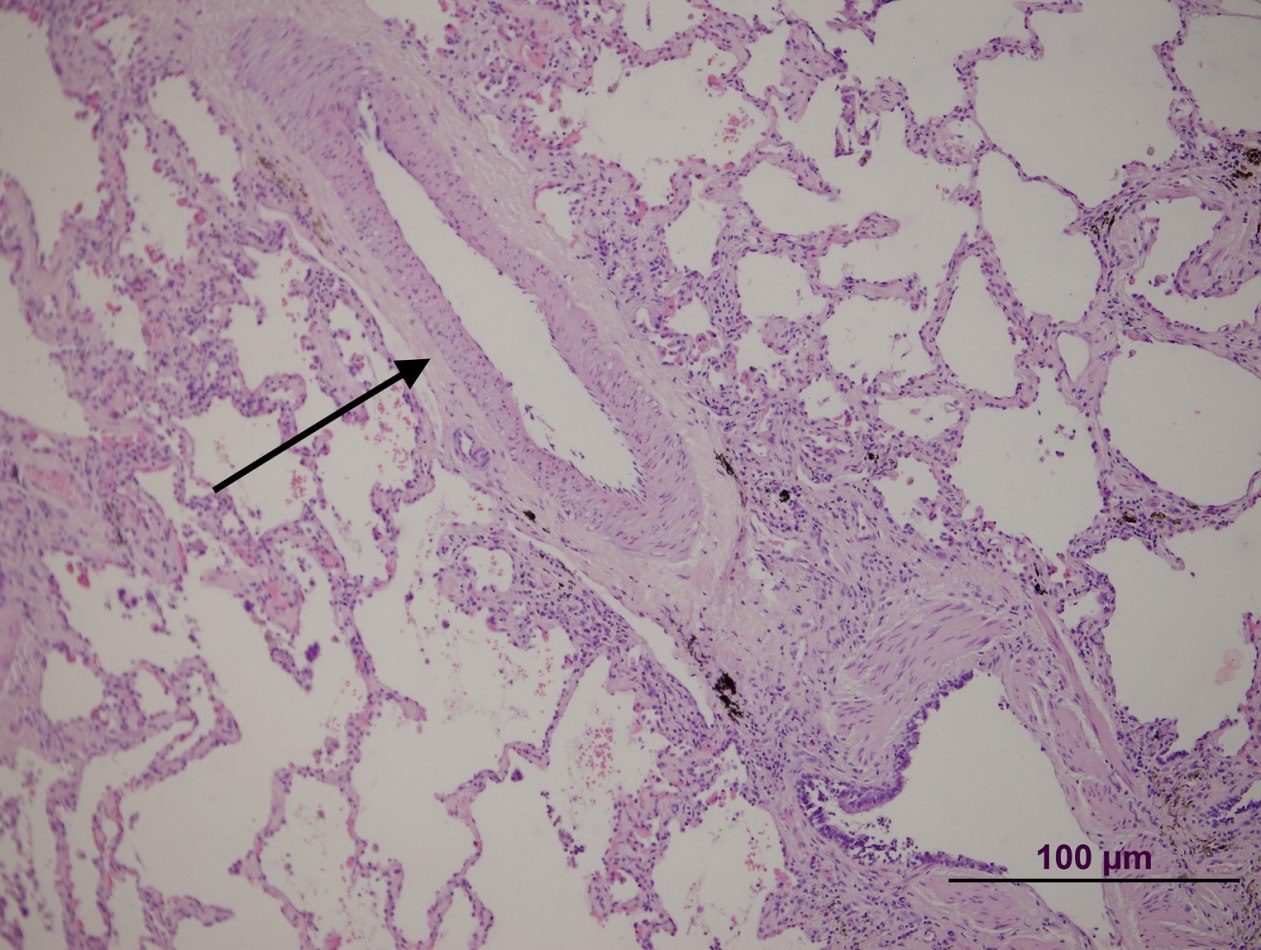
Patient 1 lung biopsy. Pulmonary artery without significative lesions (arrow).

**Fig 4:**
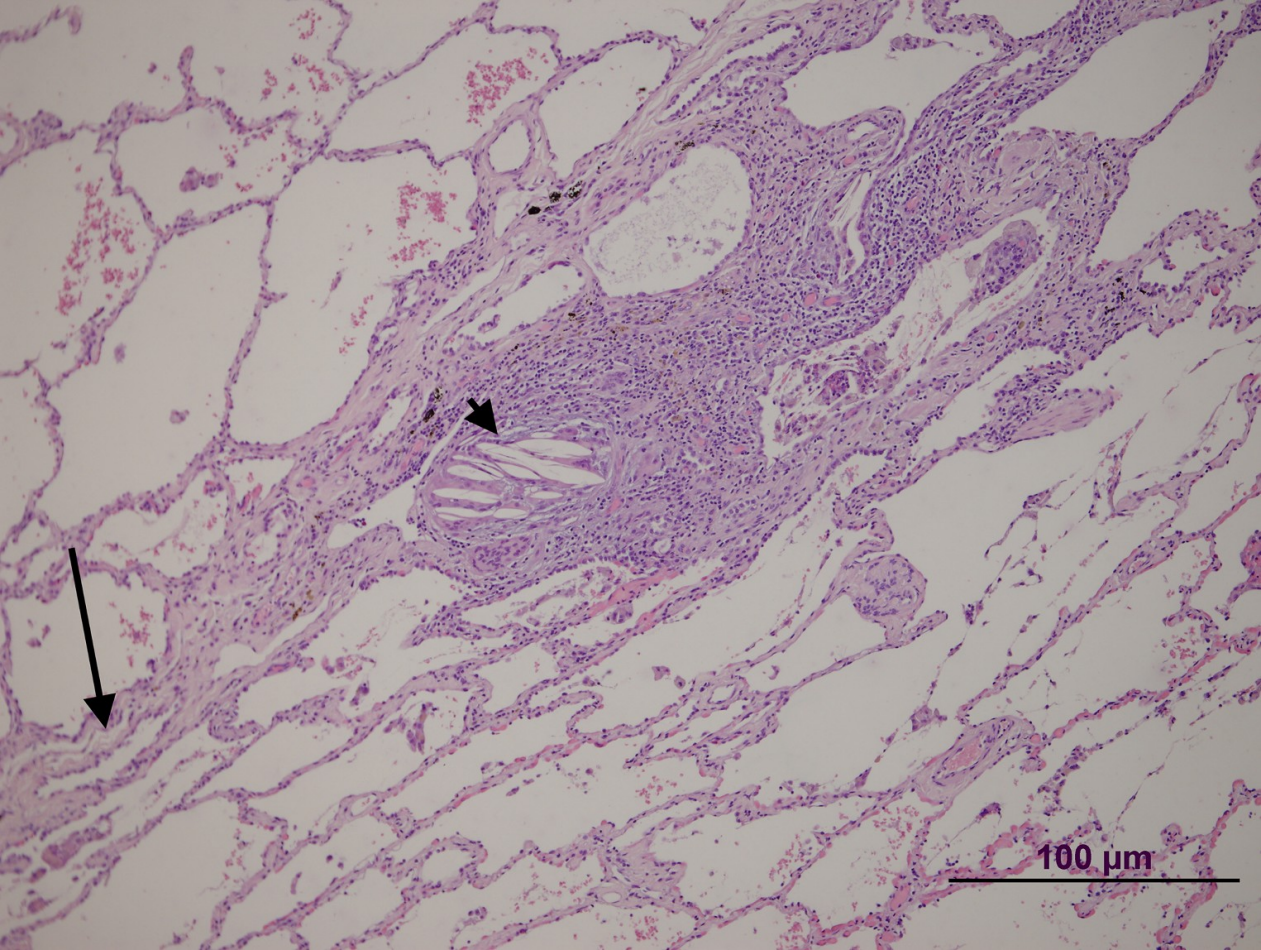
Patient 1 lung biopsy. Peripheral area. A septum is shown (arrow) with a vein without significant changes. Note the granuloma and giant cells with prominent intracytoplasmic cholesterol clefts (arrowhead).

**Fig 5:**
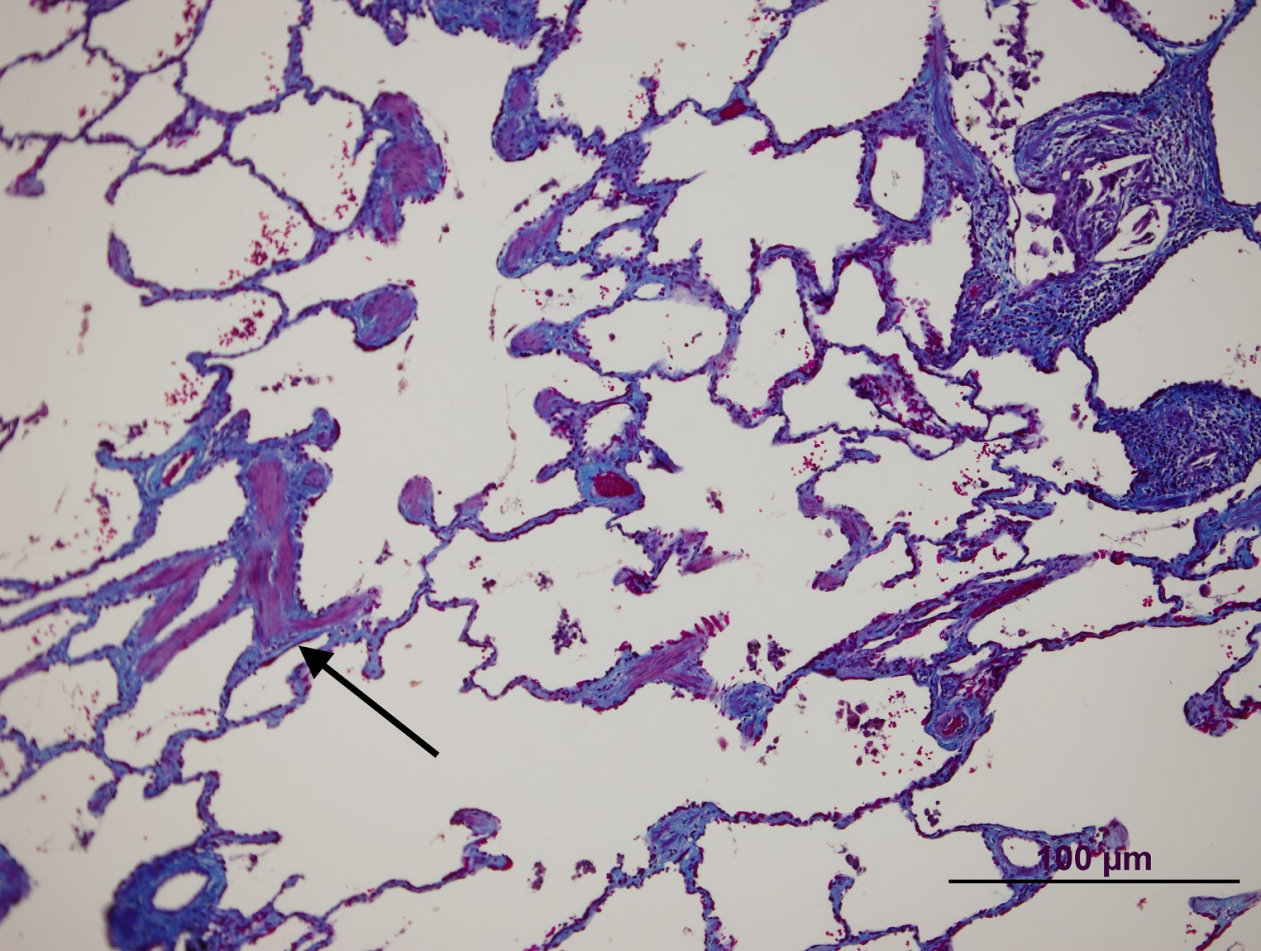
Patient 1 lung biopsy. Masson Trichrome staining showing an emphysematous change with muscle cell hyperplasia (arrow).

**Fig 6:**
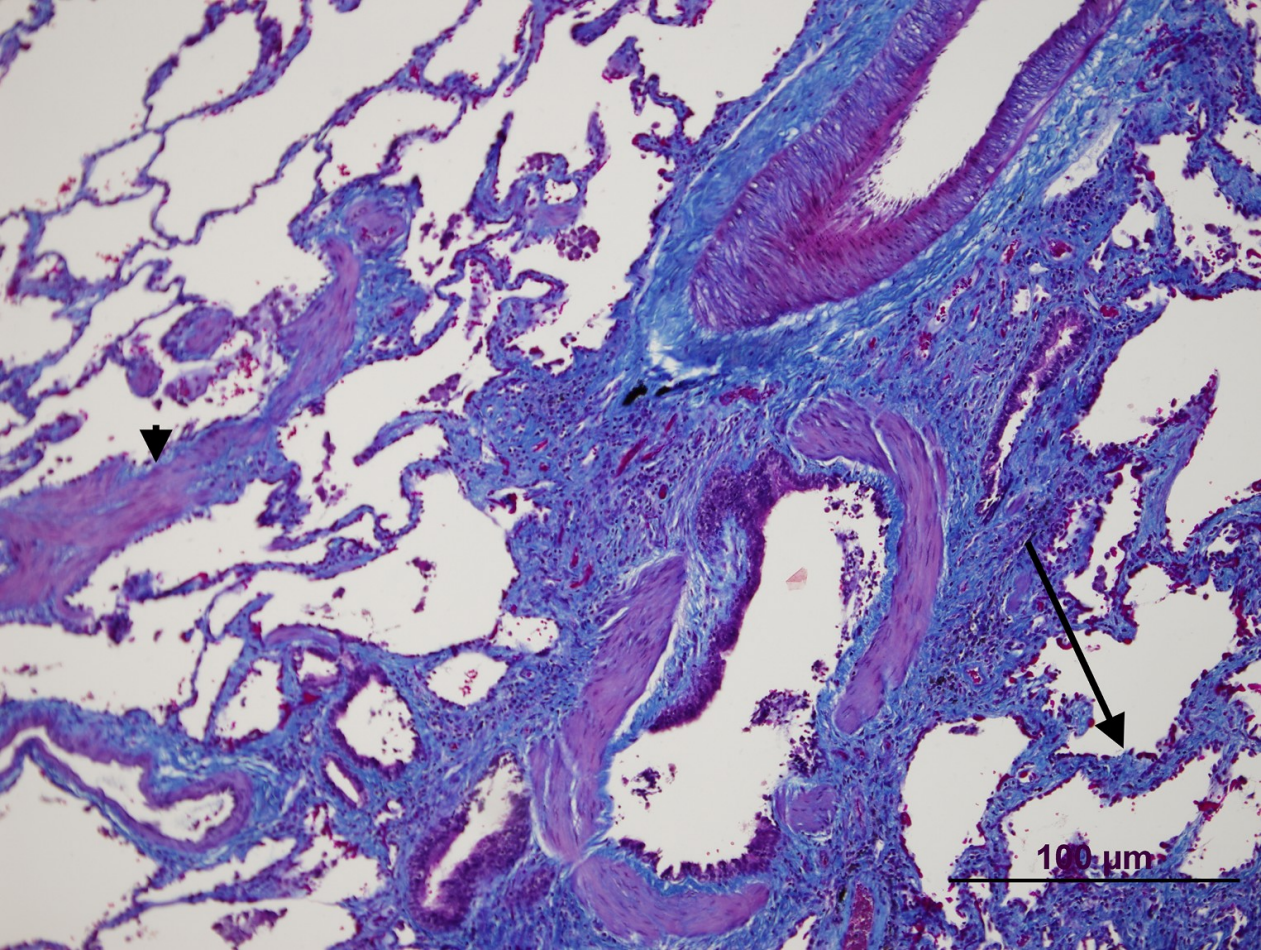
Patient 1 lung biopsy. Masson Trichrome staining showing pulmonary fibrosis (arrow) near the airway. Note also muscle cell hyperplasia (arrowhead).

These two patients were diagnosed at 26 and 59 years of age, respectively. Patient 1 was initially diagnosed with Interstitial Lung Disease. In the MDCT, she had interlobular septal thickening and emphysema radiological pattern with peripheral distribution (Fig 2). In the initial diagnostic workup, she underwent a lung biopsy, which confirmed Non-specific Interstitial Pneumonia, with fibrotic pattern and the presence of peribronchial and perivascular granulomas (Figs 3–6). Pulmonary Function Test showed a mild restrictive pattern (Total Lung Capacity (TLC) 74% of predicted) and severe diffusion capacity reduction (Diffusing Capacity of the Lung for Carbon Monoxide (DLCO) 37% and Coefficient for Carbon Monoxide (KCO) 67% of predicted, respectively). Immunosuppressive therapy was prescribed with azathioprine. During follow-up, goal-oriented PAH therapy was applied and risk profile assessed periodically. Currently, she receives systemic prostanoids and has been referred to the lung transplant unit for evaluation.

Patient 2 does not have radiological signs suggestive of interstitial lung disease. The only remarkable finding was a subtle emphysema pattern. She is also in treatment with systemic prostanoids.

### Family 2

**Fig 7:**
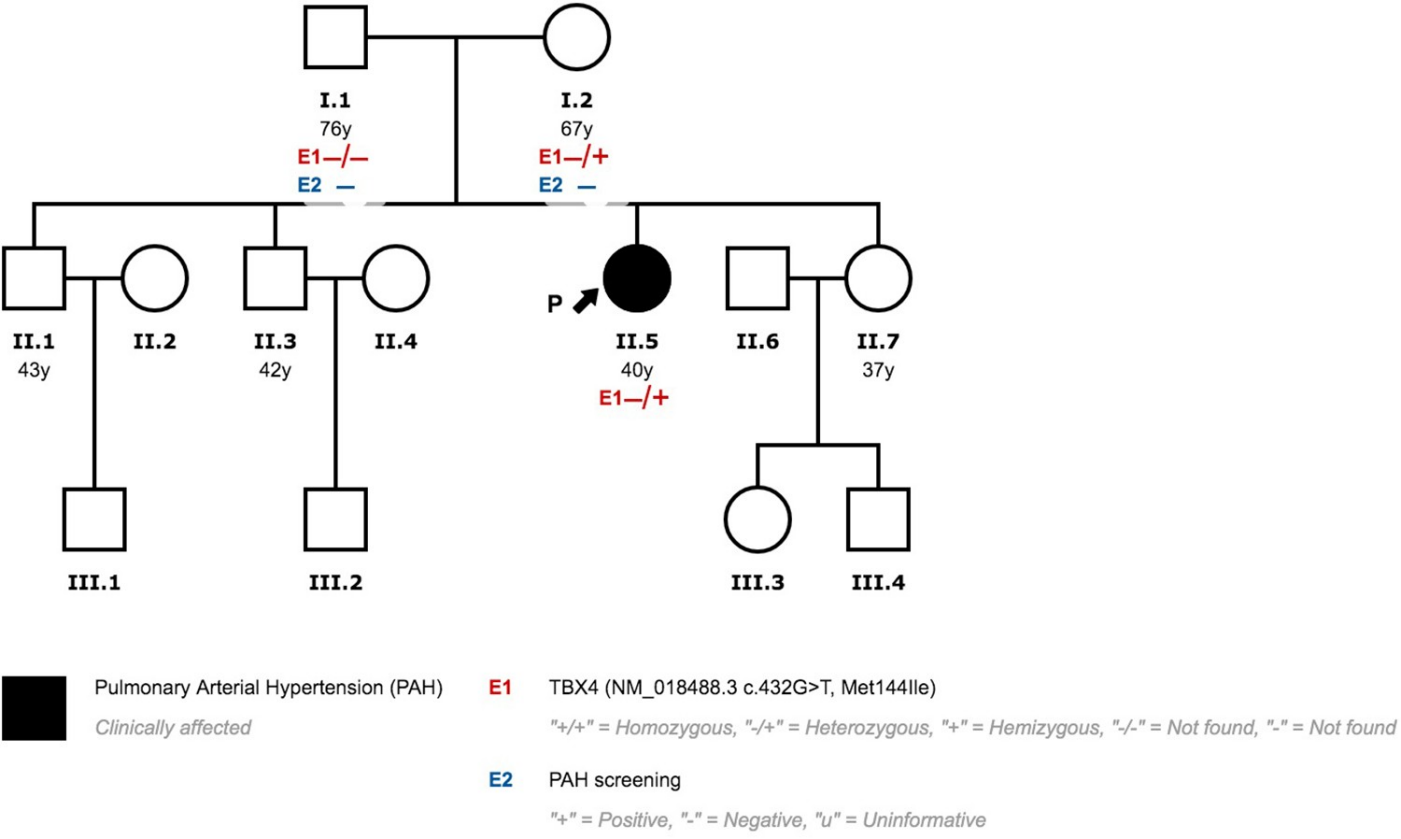
Family 2 pedigree.

Patient three presented a missense variant (NM_018488.3:c.432G>T; p.(Met144Ile). This variant has not been previously published in the literature and showed an extremely low frequency in the control population (gnomADexomes: 4,06x10-6, gnomAD genomes: 0, Kaviar: 0, EVS:0). On gnomADexomes v2.0.2 there is only one carrier in the Latino population (1/246213). The phenotype of this subject is unknown. The majority of the bioinformatic pathogenicity prediction tools suggested a pathogenic effect (8/10). The prevalence of this variant in affected individuals is significantly increased when compared with controls. Following ACMG guidelines, we consider this variant likely pathogenic [21]. The family screening revealed that her mother also carried the variant. After a comprehensive study, PAH was ruled out. Genetic Counseling was provided to the rest of the family (Fig 7).

**Fig 8:**
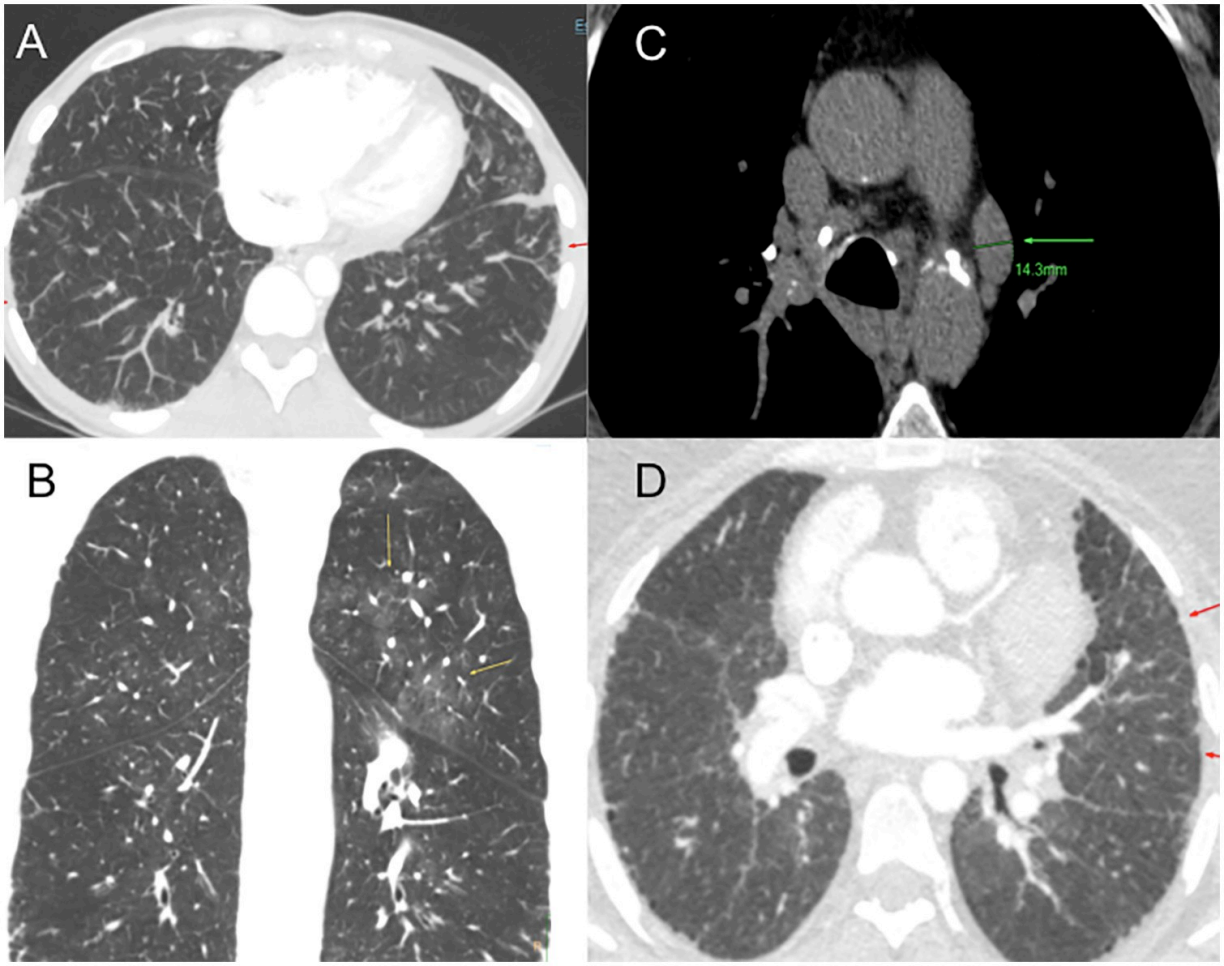
Multidetector Computed Tomography patients 3 and 4. (A) Patient 3 interlobular septal thickening (red arrow) (B) Patient 3 ground grass pattern (yellow arrow) (C) Patient 4 mediastinal lymphadenopathies (green arrow) (D) Patient 4 interlobular septal thickening (red arrows).

**Fig 9:**
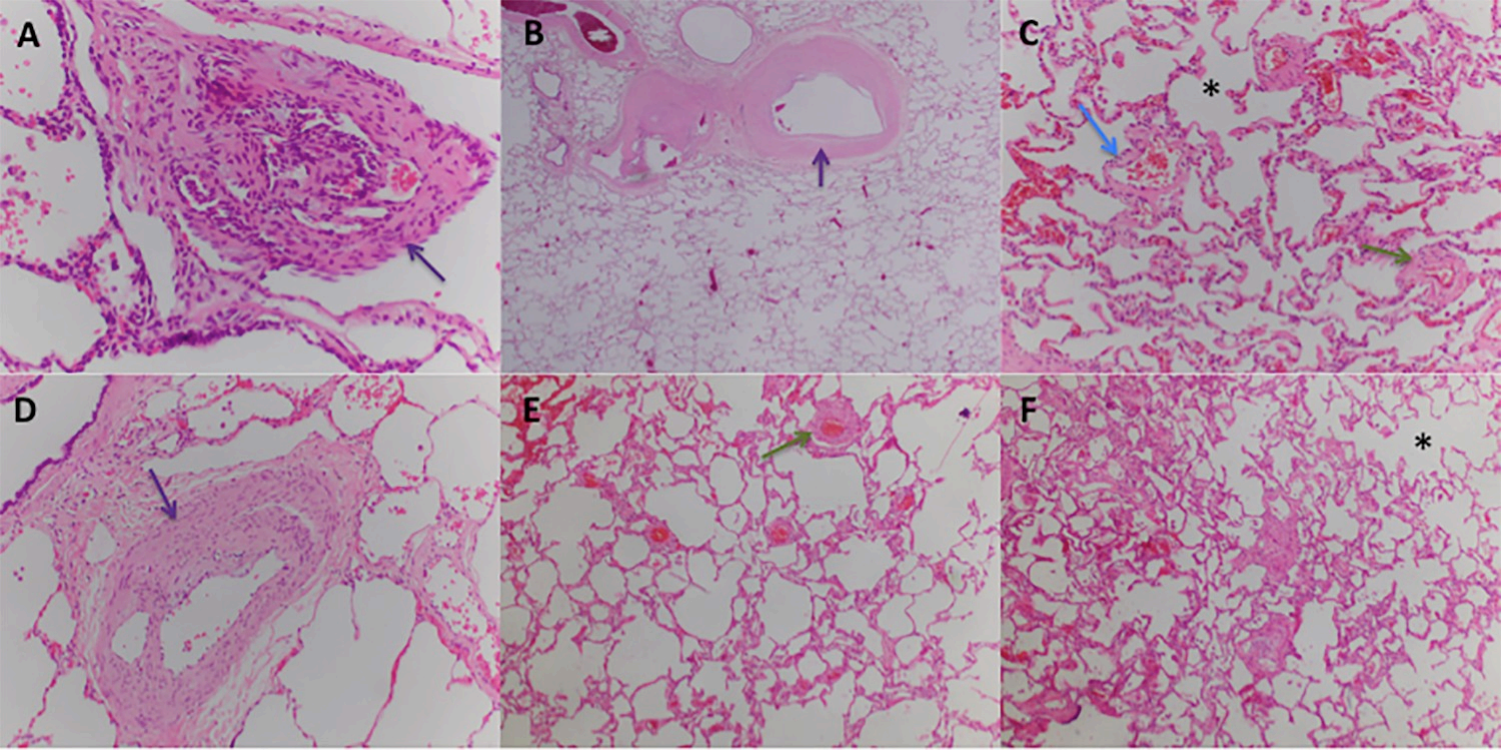
Patient 3 explanted lung tissue. Typical PAH findings, without venous or venular lesions. (A) Vascular structure with glomeruloid morphology. 10X view H&E stain. (B) Artery intimal thickening (arrow). 4X view H&E stain. (C) Lung parenchyma with emphysema signs with dilated alveolar spaces and incomplete alveolar septa (star), increase in vascularization and capillary dilation (blue arrow) and artery medial hypertrophy (green arrow). 4X view H&E stain. (D) Artery intimal thickening (arrow). 10X view H&E stain. (E) Artery medial hypertrophy (green arrow) (F) Dilated alveolar spaces (star). 4X view H&E stain.

Patient 3 is a Caucasian female diagnosed with PAH at 28 years of age. She was clinically diagnosed with possible PVOD due to radiological findings and reduced diffusion capacity. At diagnosis, oral therapy with Phosphodiesterase 5 inhibitors (PDE5i) and Endothelin Receptor Antagonist (ERA) was prescribed. Initially, the patient responded favourably to vasodilator treatment. Six years after diagnosis, she progressively worsened and treatment had to be escalated. Systemic prostanoids had to be added and treprostinil was titrated up to optimal dose. She did not develop pulmonary oedema after treatment with prostanoids. Despite optimal medical treatment, she presented a high risk of death and was listed for lung transplantation. Nine years after diagnosis, she underwent bilateral lung transplantation. MDCT study at diagnosis showed subtle ground grass and septal thickening (Fig 8). Histological analyses after transplantation reveal typical PAH findings (Fig 9). PVOD and Interstitial Lung Disease were also ruled out in explanted lung tissue (Fig 9).

### Family 3

**Fig 10:**
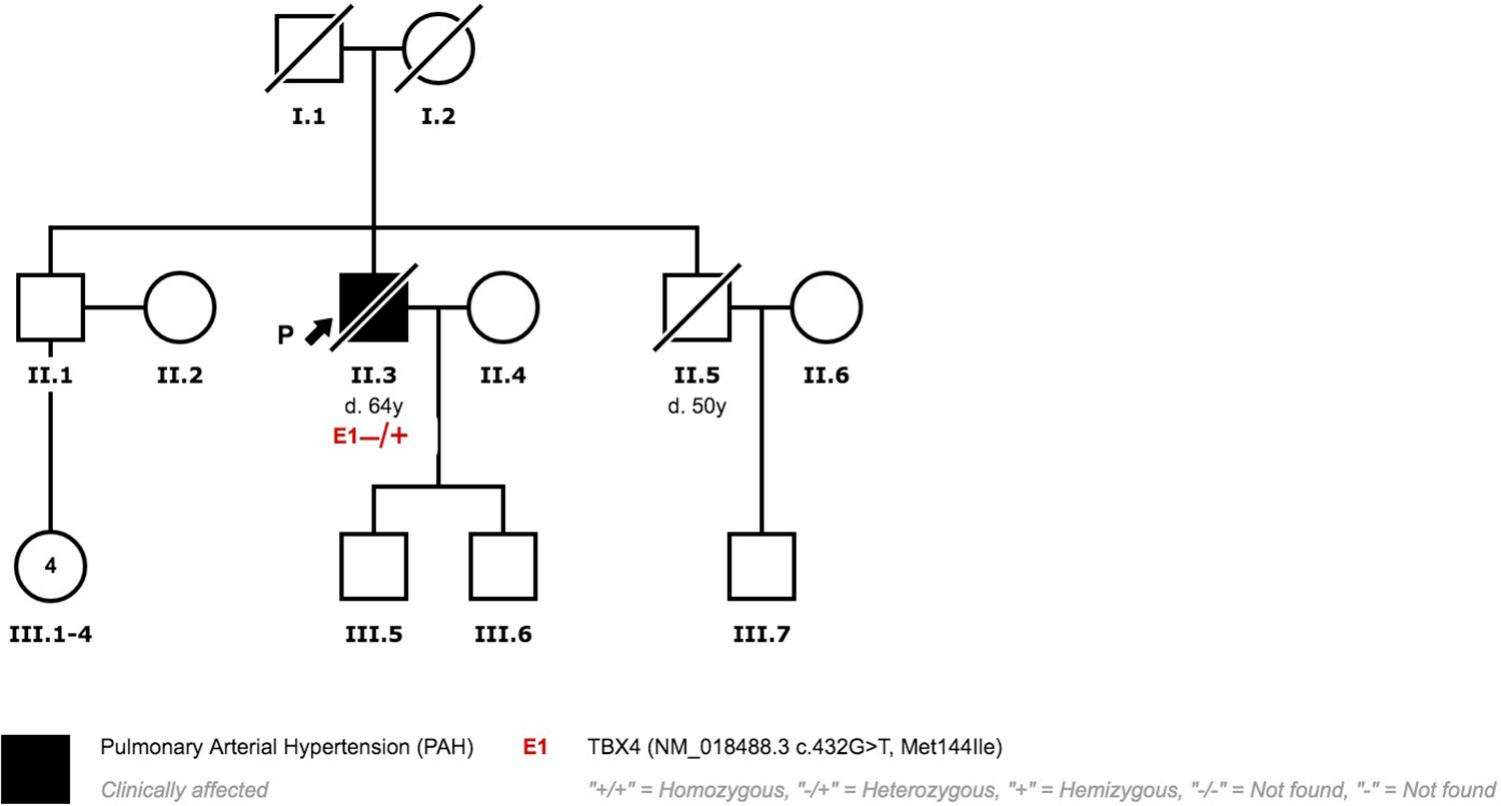
Family 3 pedigree.

Patient 4 also presented the same missense variant (NM_018488.3: c.432G>T; p.(Met144Ile)) documented in patient 3 (See Above). Patients 3 and 4 are not related and came from different regions of the country. Patient 4 parents’ had died before PAH diagnosis and before genetic tests could be offered. Genetic counselling has been provided to the rest of the family. The rest of the family members rejected genetic testing (Fig 10).

Patient 4 was a Caucasian male diagnosed with PVOD at 62 years of age. Initially, he displayed poor clinical status, showing heart failure signs and severe respiratory insufficiency. The radiological study was highly suggestive of PVOD with the typical triad (Fig 5) [14]. Referred to the lung transplant unit, he was not eligible due to advanced age and comorbidities. The clinical course was rapidly progressive and he died 26 months after diagnosis.

### Family 4

**Fig 11:**
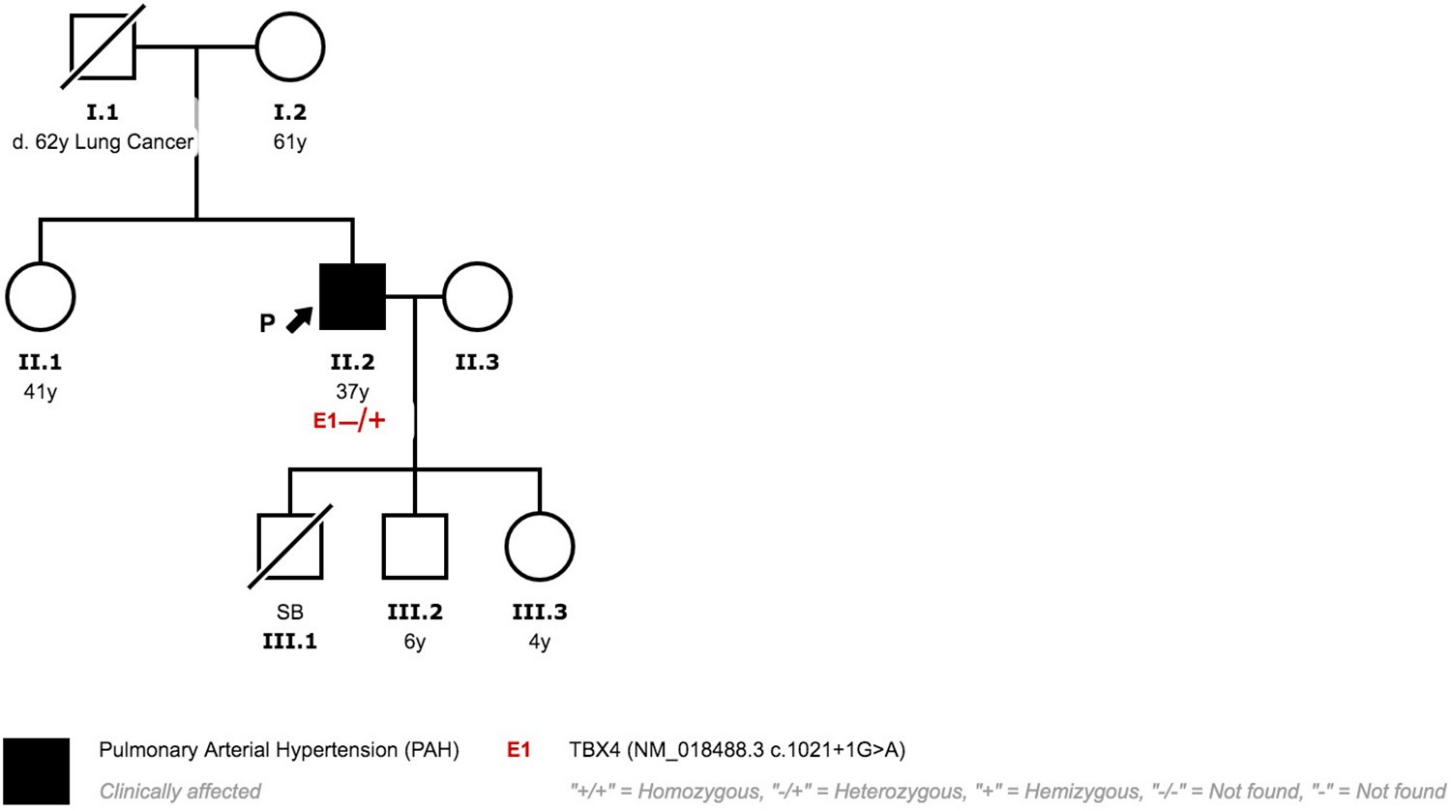
Family 4 pedigree.

Patient 5 showed a splicing variant (c.1021+1 G>A), not previously published. The majority of the pathogenic bioinformatics tools predicted a pathogenic effect for the variant. The splicing prediction software also showed a possible effect on splicing with a new possible cryptic splice site at +30bp. This variant has not been found in the analyzed control population databases (Table 2). Functional analysis of this variant by minigenes assay confirmed the alternative splicing with exon 6 and 7 skippings (data in publishing). The variant was classified as pathogenic following ACMG guidelines [21]. No other variants were observed in the rest of the genes included in the panel, including genes related to Hereditary Hemorrhagic Telangiectasia (ENG, ACVLR1). The patient had a son who died in the perinatal period due to severe PH and multiple vascular malformations. Cascade screening was rejected by the other family members (Fig 11).

**Fig 12:**
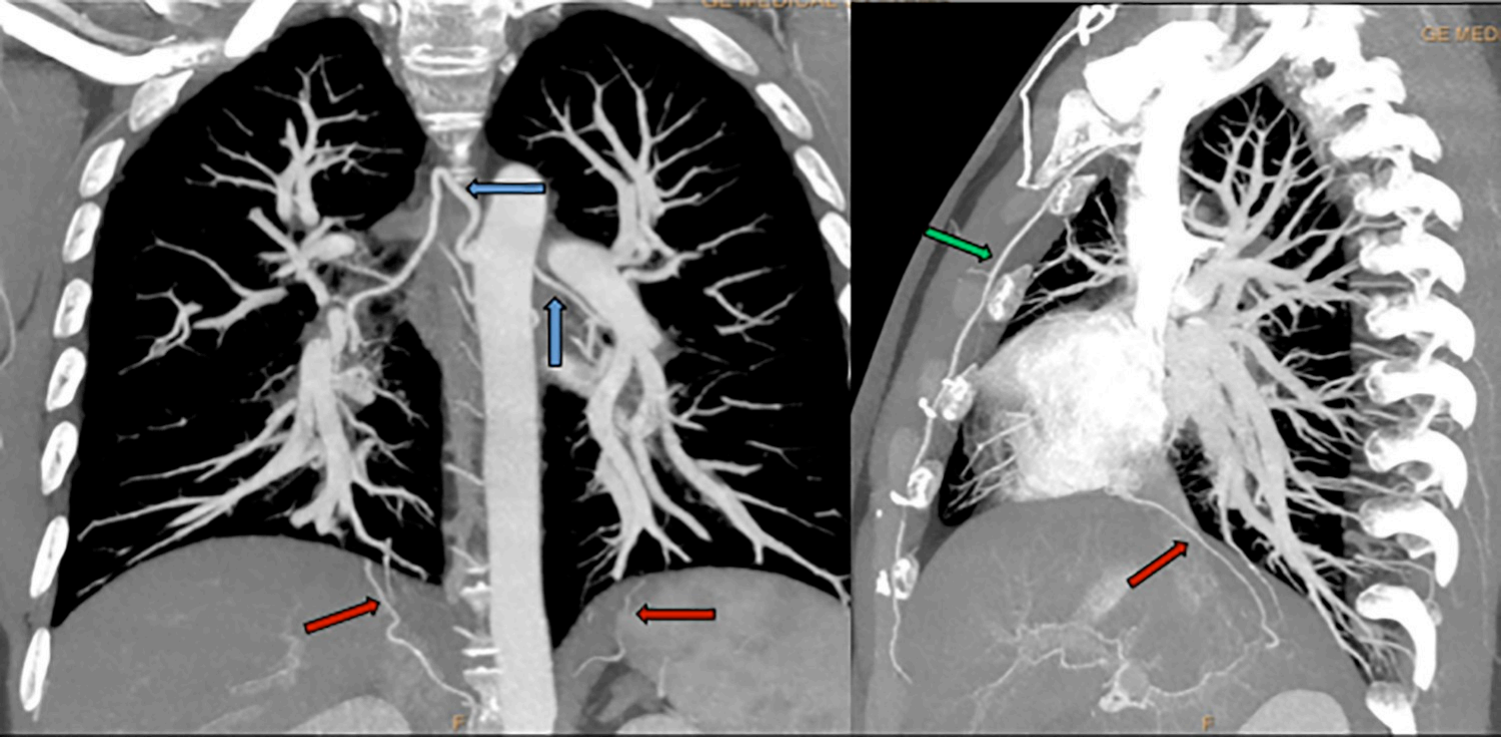
Multidetector Computed Tomography patient 5. Hypertrophy in mammary (green arrows), phrenic (red arrows) and bronchial arteries (blue arrows).

Patient 5 was diagnosed with PAH at 31 years of age. Hereditary Hemorrhagic Telangiectasia (HHT, Osler-Weber-Rendu syndrome) was initially suspected. In the CT scan at diagnosis, he had marked collateral circulation with hypertrophy in mammary, phrenic and bronchial arteries (Fig 12). In the RHC, a high cardiac output was observed. A comprehensive study was performed to find arteriovenous malformations in other sites and they were not observed. Due to the lack of other clinical findings, HHT was not confirmed. In the initial RHC, he had a positive acute response in the vasoreactivity test. However, we observed unresponsiveness to long-term Calcium Channel Blockers (CCB) and PAH-targeted treatment had to be added.

### Family 5

**Fig 13:**
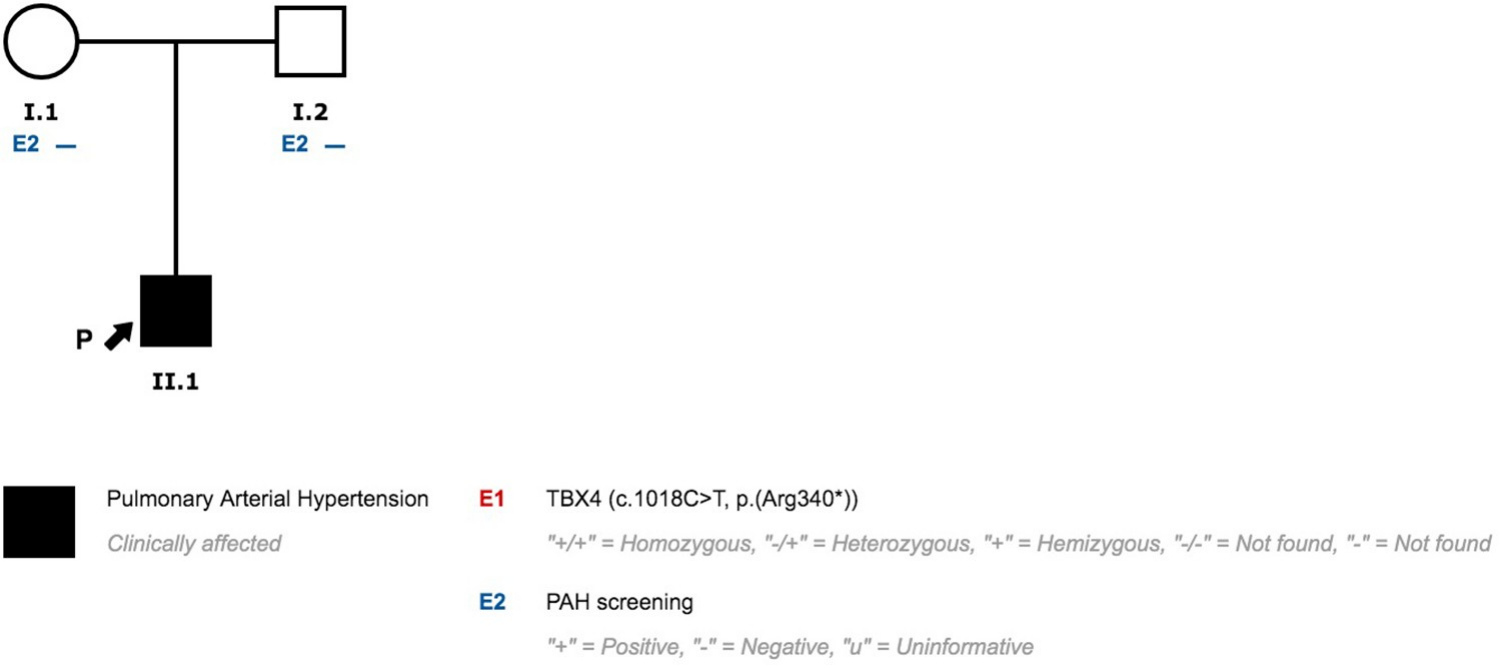
Family 5 pedigree.

Patient 6 showed a nonsense mutation in *TBX4* (*TBX4*:NM_018488.3:c.1018C>T:p.(Arg340*)). The variant has previously been reported by Galambos et al. and classified as Likely Pathogenic [8]. He also carries a variant in exon 4 of gene *SMAD1* (c.738G>C(p.Met246Ile)). This sequence change replaces methionine with isoleucine at amino acid 246. The variant has been found at extremely low frequency or is absent in the analyzed control population databases (gnomAD exomes, gnomAD genomes, Kaviar, ESP, 1000G, Beacon). Most pathogenic bioinformatics tools suggested that this variant impaired the function of the encoded protein. Following ACMG guidelines, this variant was considered of unknown significance [21]. Genetic counselling has been provided to the rest of the family. However, cascade screening was rejected (Fig 13).

Patient 6 was first diagnosed with PH in childhood and referred to our center at 24 years of age. Secundum Atrial Septal Defect (ASD) was observed in the initial diagnostic workup. The larger diameter was 2 cm and right-to-left shunting was present. Severe PAH was confirmed in the RHC. Considering these findings, ASD closure was contraindicated. It is also remarkable a moderate reduction in diffusion capacity (58% of predicted) and severe respiratory insufficiency. Ambulatory oxygen therapy was initiated after diagnosis. Due to the clinical situation, combination therapy was initiated and a goal-oriented strategy was applied. Two years after diagnosis, inhaled iloprost was initiated and currently, he receives systemic prostanoids.

### Family 6

**Fig 14:**
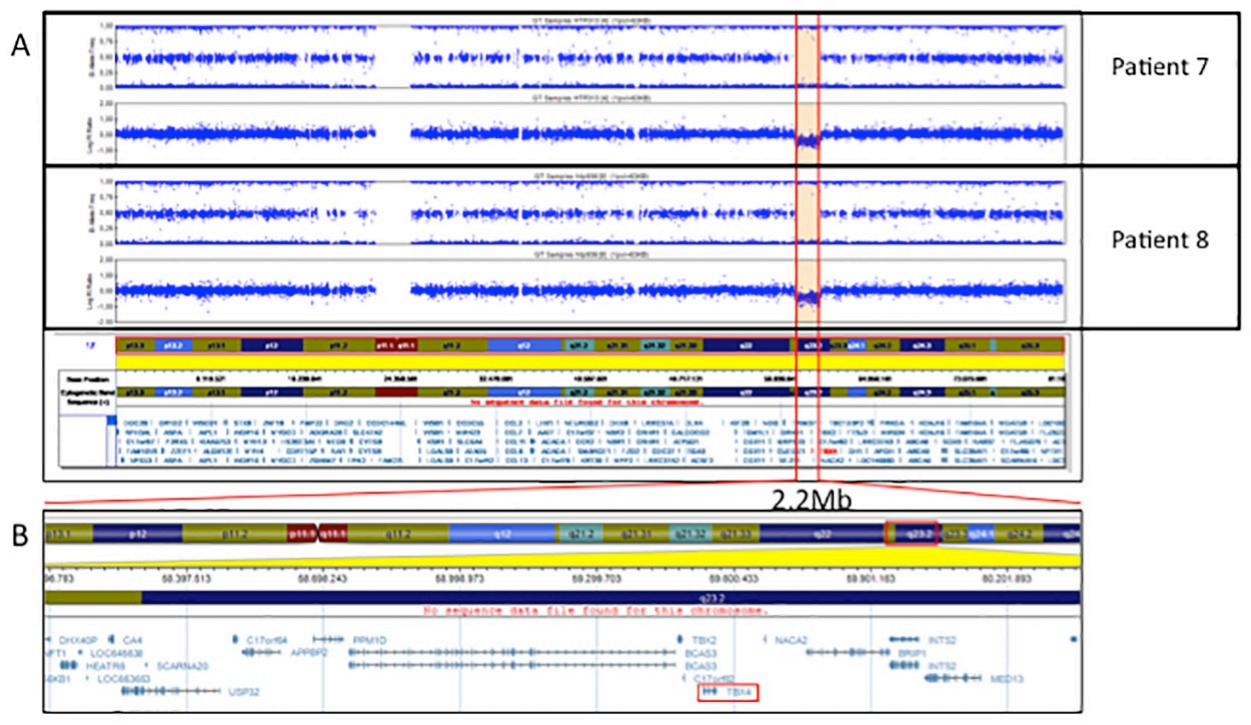
SNP microarray validation of detected deletion through NGS in patients 7 and 8.

**Fig 15:**
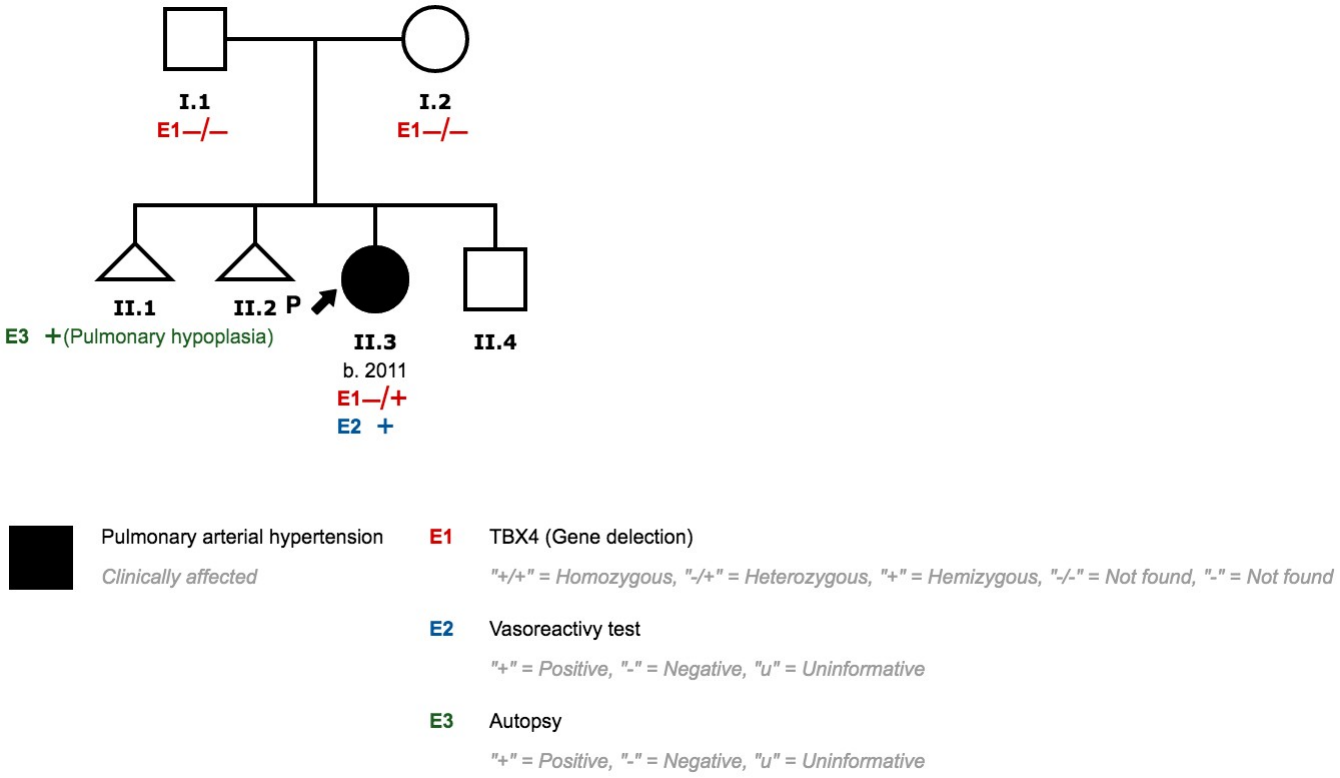
Family 6 pedigree.

We identified a 2.2 MB CNV deletion involving TBX4 in patient 7, which encompasses 17 genes and potential genes (*CA4*, *LOC645638*, *HEATR6*, *LOC653653*, *SCARNA20*, *USP32*, *C17orf64*, *APPBP2*, *PPM1D*, *TBX2*, *BCAS3*, *C17orf82*, *TBX4*, *NACA2*, *BRIP1*, *INTS2* and *MED13*) (Fig 14). She was diagnosed with Persistent Pulmonary Hypertension of the Newborn (PPHN) at the age of 4 months. Her mother had previously had two spontaneous abortions. The histological analysis of one of them showed pulmonary hypoplasia. Both parents are healthy. PAH and lung disease were ruled out in her brother (Fig 15).

In imaging studies, there was no evidence of parenchymal lung disease. She had bilateral nystagmus, which required surgery. She also showed mild growth and neurodevelopmental delay. At diagnosis, dual oral therapy was initiated with ERA and iPDE5 until she was five years old. RHC was repeated and we observed a positive response to the vasoreactivity test. A Calcium Channel Blocker was added and long-term response to CCB was confirmed with near normalization hemodynamic parameters (Indexed PVR 3.1 UW.m2 and mPAP 18 mmHg).

### Family 7

**Fig 16:**
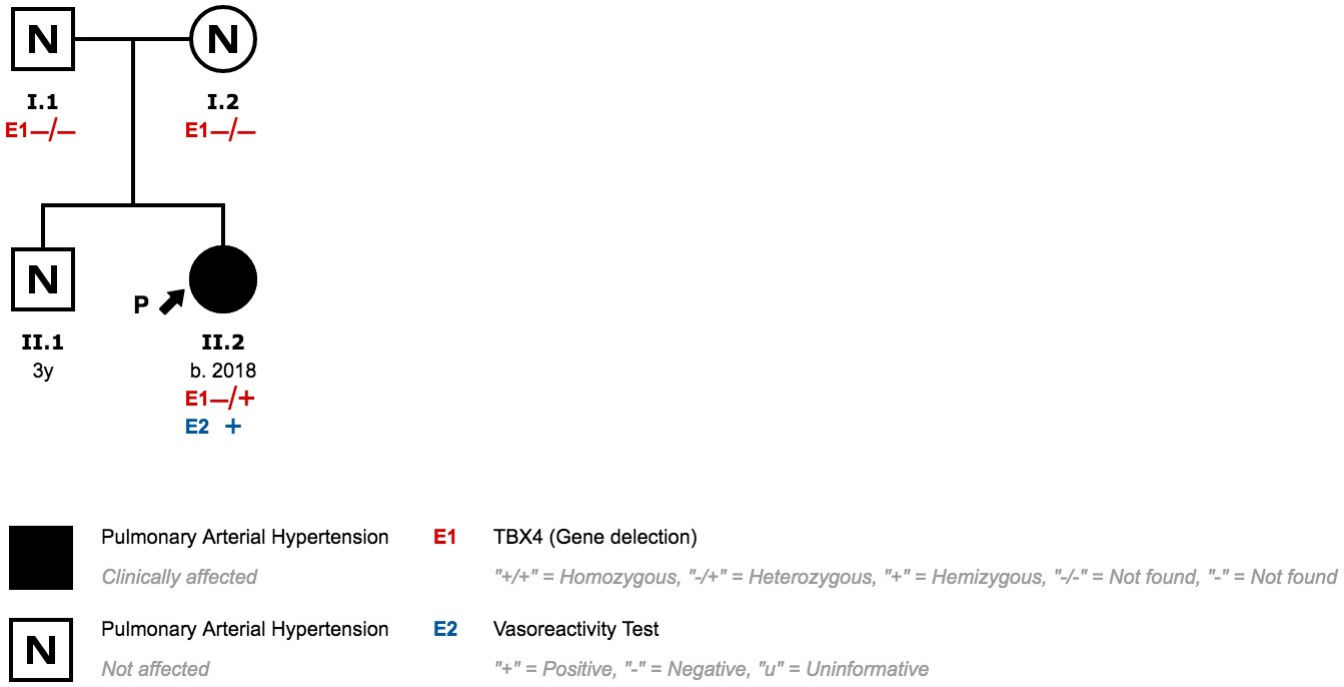
Family 7 pedigree.

The same 2.2Mb CNV observed in patient 7 was detected in patient 8. She was diagnosed with suprasystemic PAH 8 days after birth. Both parents and her 3-year-old brother have no signs of PAH or lung disease (Fig 16).

**Fig 17:**
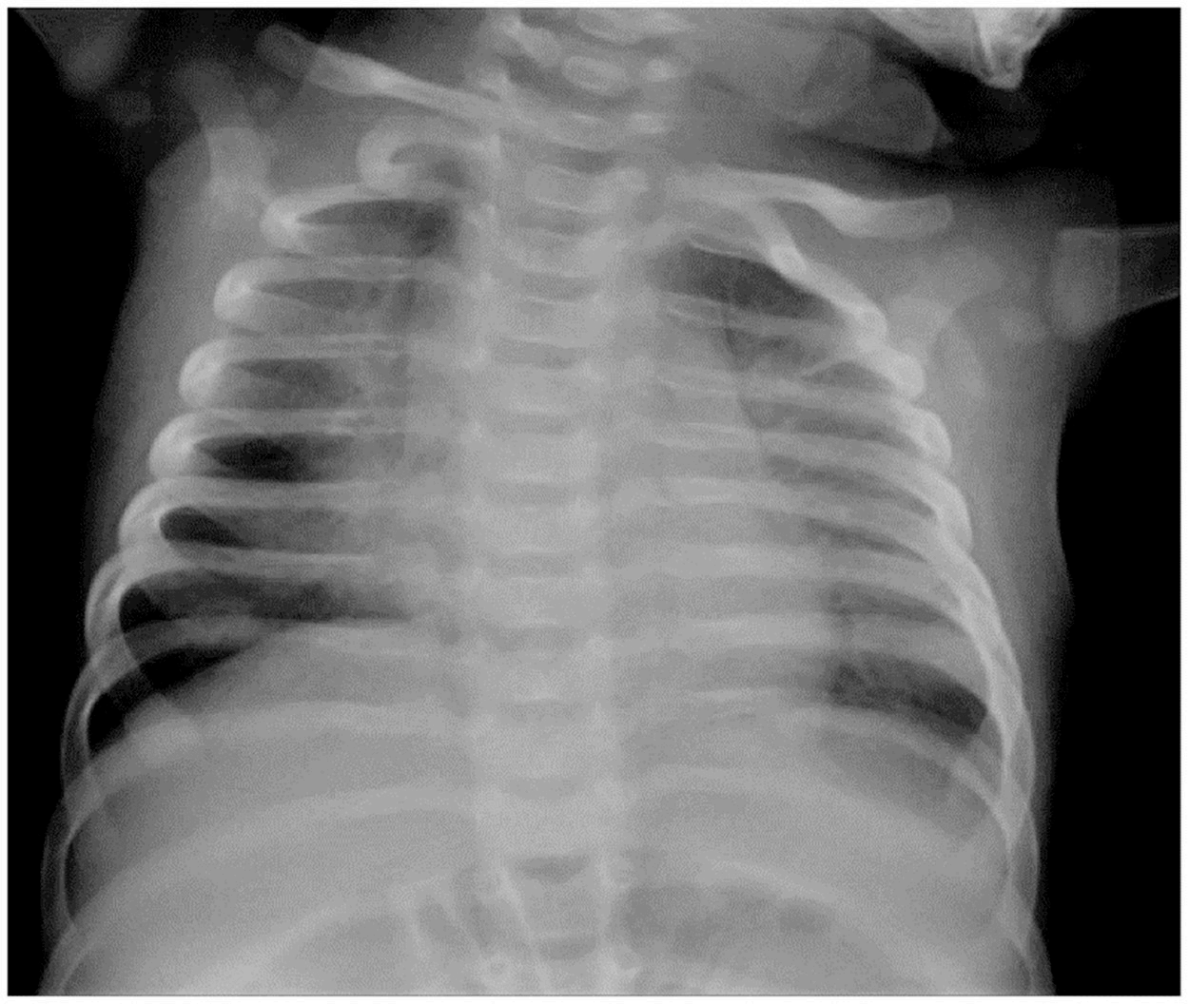
Patient 8 chest X-ray at 4 months.

She was initially treated with sildenafil and the hemodynamic situation was reassessed at 4 months of age. In the RHC, severe precapillary PH was confirmed and we observed partial positive response to acute vasodilator test. Treprostinil was initiated and titrated up to medium dose. She responded favourably to PAH-targeted treatment with clinical improvement and proper growth. Imaging studies showed Patent Ductus Arteriosus and Patent Foramen Ovale. She also had radiological signs of Interstitial Lung Disease and initially needed oxygen (Fig 17).

## 4. Discussion

In this study, we describe our experience with PAH possibly associated with *TBX4* variants. The most important finding is a moderate to a severe reduction in diffusion capacity in all adult-onset patients. Apart from this, we observe a wide spectrum of clinical phenotypes, which have not been previously described.

A diffusion capacity reduction in *TBX4*-associated PAH is a novel finding. A recent study by Gräf et al. studied a large PAH cohort, which included 1048 patients [26]. Fourteen of them carried a pathogenic variant in the *TBX4* gene, with a mean age of 56.5 years (39.9–71.8 years). In this group of patients, diffusion capacity was not reduced and the mean KCO was 89.7% (83.3-91-8%).

*TBX4* variants are a common cause of hereditable PAH in infants and children. Pediatric PAH due to *TBX4* mutations is typically associated with musculoskeletal disorders, congenital heart disease and intellectual disability [7–11]. Kerstjens-Frederikse et al. first described *TBX4* mutations or deletion in 6 children with PAH and Small Patella Syndrome [7]. A recent study assessed clinical and histological data in 19 children carrying *TBX4* variants [8]. These patients had Persistent Pulmonary Hypertension in Neonates (PPHN), childhood-onset PH and lung development abnormalities. A distinctive biphasic pattern was observed in 12 /19 infants. This pattern consists of PPHN or transient respiratory distress with improvement or resolution, followed by PH diagnosed in the pediatric period (median age 1.5 years).

However, a typical phenotype has not been described in an adult population. In our study, a common finding in adult-onset patients with PAH carrying possibly associated pathogenic variants in *TBX4* is a moderate to severe DLCO reduction. This is a typical finding in PAH associated with scleroderma, PVOD or PH from group 3 [14, 27, 28]. In PH from Group 1, a decreased DLCO is associated with advanced age, male, reduced peak oxygen uptake and higher Functional Class. Furthermore, previous studies suggest DLCO as a prognostic marker in PAH [29, 30].

In our study, we observed eight patients with PAH possibly associated with *TBX4* variants and four healthy carriers. This incomplete penetrance is a hallmark of hereditable PAH. The presence of a pathogenic variant is required but insufficient alone for clinical expression. Furthermore, this study highlights variable expression both within and between families. Patients 1–2 and patients 3–4 carry the same variant, respectively. However, age at diagnosis, clinical phenotype and prognosis are completely different in each patient. Additional genetic or environmental factors may affect the clinical expression. The different clinical phenotypes observed in our population are described below.

Despite an ever-growing knowledge of PAH, the differential diagnosis between PAH and PVOD remains a clinical challenge [18, 31]. Hadinnaopla et al. have recently demonstrated that genetic testing is a useful tool in the differential diagnosis in patients with overlapping clinical features [17]. In this study, up to 36% PVOD patients were initially diagnosed with PAH and reclassified after genetic testing.

In our study, PVOD was suspected in two patients based on imaging, severe respiratory insufficiency and diffusion reduction. Patient 3 was treated with PAH targeted therapy, including high doses of systemic prostanoids. Despite the suspicion she responded favourably, with clinical and functional stabilization, making the PVOD diagnosis unlikely. Genetic testing subsequently ruled out *EIF2AK4* mutations and histological analysis showed no signs of PVOD. On the other hand, patient 4 had an aggressive form and a rapidly progressing course. Given the initial suspicion of PVOD, PAH targeted therapy was prescribed with caution and no systemic prostanoids were administered. This conservative management, clearly determined initial suspicion of PVOD, might have influenced the progressive clinical course observed.

These findings reflect the complexity of phenotyping PH. PVOD-like involvement is a major issue in PAH. Although a high probability diagnosis of PVOD can be achieved by the combination of clinical features, pulmonary function test and radiological findings, a definitive diagnosis can only be established by histological or genetic analysis. Clinical genetic testing is of value in the early identification of PVOD. However, as previously reported by our group, the prognosis is mainly determined by the tolerance to pulmonary vasodilator independently of the presence or absence of an underlying biallelic *EIF2AK4* mutation [15]. Furthermore, a favourable clinical response to PAH therapy runs against classic PVOD.

*TBX4* pathogenic variants can also lead to lung lethal lung developmental disorders. Karolak et al. have recently published a large series of 26 neonate deceased patients with acinar dysplasia, congenital alveolar dysplasia and other rare lung hypoplasia due to disruption of the TBX-FGF pathway [13]. They identified rare heterozygous copy-number variant deletions or single-nucleotide variants involving *TBX4* in 8 and 2 patients, respectively. Furthermore, different genes have been associated with Idiopathic Interstitial Pneumonia. The majority of the pathogenic variants were identified in the surfactant genes *SFTPA1*, *SFTPA2*, *SFTPB*, *SFTPC*, *ABCA3*, and *NKX2*-1 [32].

In our series, patient 1 was diagnosed with Non-Specific Interstitial Pneumonia. She underwent lung biopsy due to clinical and radiological findings. In the histological analysis, interstitial fibrosis without temporal heterogeneity and cholesterol granulomas were present. Pulmonary Function Testing was mildly impaired and radiological parenchyma abnormalities were only modest. These, together with the *TBX4* pathogenic variant, are criteria favouring Group 1 PH. Contextualizing PH severity, pulmonary hypertension cannot be easily explained by the presence of a mild form of lung disease. The most plausible interpretation is that the patient suffers from hereditable PAH associated with a *TBX4* variant and probable parenchyma lung disease. These findings are in line with a case recently published by Maurac et al. [33]. They report a patient with parenchymal pulmonary abnormalities, airway diverticula, small patella syndrome, and PAH. Despite the lung pathology, PAH was the most plausible diagnosis. Furthermore, Eyries et al. have recently shown a similar phenotype in PAH associated with *KDR* mutations [34]. They describe two PAH patients with mild Interstitial Lung Disease and low DLCO who carried a loss-of-function mutation in the *KDR* gene. In one family, segregation analysis revealed that the variant was associated with reduced DLCO, with or without PAH. Further investigations are needed to address the relation between *TBX4* or other PAH genes and interstitial lung diseases.

Patient 5 had anomaly enlarged systemic arteries and high cardiac output at diagnosis. Hereditary Hemorrhagic Telangiectasia was initially suspected. However, typical Pulmonary Arteriovenous Malformations were not present and HHT was not confirmed. Furthermore, no variants were observed in genes associated with HHT (*ENG* and *ACVRL1*). His son died on the first day of life due to a severe form of pulmonary vascular malformation and pulmonary hypertension. The infant probably had a different clinical manifestation associated with *TBX4* variants.

Patients with HAP often show Bronchial Arteries (BA) enlargement, which might reflect the role of preexisting Intrapulmonary Bronchopulmonary Anastomoses (IBA) [35, 36]. An increase in Pulmonary Artery resistance might lead to an increased flow toward an open IBA and, subsequently, Bronchial Arteries. Furthermore, this phenomenon might contribute to the development of vascular plexiform lesions. Calambos et al. have provided histologic evidence supporting these findings in patients with IPAH and patients with PH associated with *TBX4* variants. This mechanism might underlie the vascular abnormalities observed in patient 5.

Patient 6 has PAH associated with a non-repaired Atrial Septal Defect. Previous studies have observed that mutations in *BMPR2* are present in up to 7.5% of patients with PAH associated with CHD [37, 38]. Our patient carries a Likely Pathogenic Variant in *TBX4* and a Variant of Unknown Significance in *SMAD1*. This gene was identified in PAH patients by Nasim et al. in 2011 [39]. The variant observed is a rare variant, found at extremely low frequency in control population databases. The role of this *SAMD1* variant in the development of PAH in this patient remains unclear. The TBX4 variant observed in our patient (c.1018C>T: p.(Arg340*)) was previously reported by Galambos et al [8]. In this study, two siblings carried this variant and also presented transient Patent Ductus Arteriosus and Patent Foramen Ovale, and Interstitial Lung Disease (ILD). During follow-up, one infant had severe ILD with mild PH and the other one ILD without PH.

These findings are interesting due to a remarkable intra- and inter-familial variable expressivity. This reflects the role of other genetic or epigenetic factors. The presence of left-to-right shunt causes an overflow in pulmonary circulation that can lead to PH. However, this does not fully explain these complex cases. Further investigation is needed to address the role of *TBX4* in CHD and ILD.

**Fig 18:**
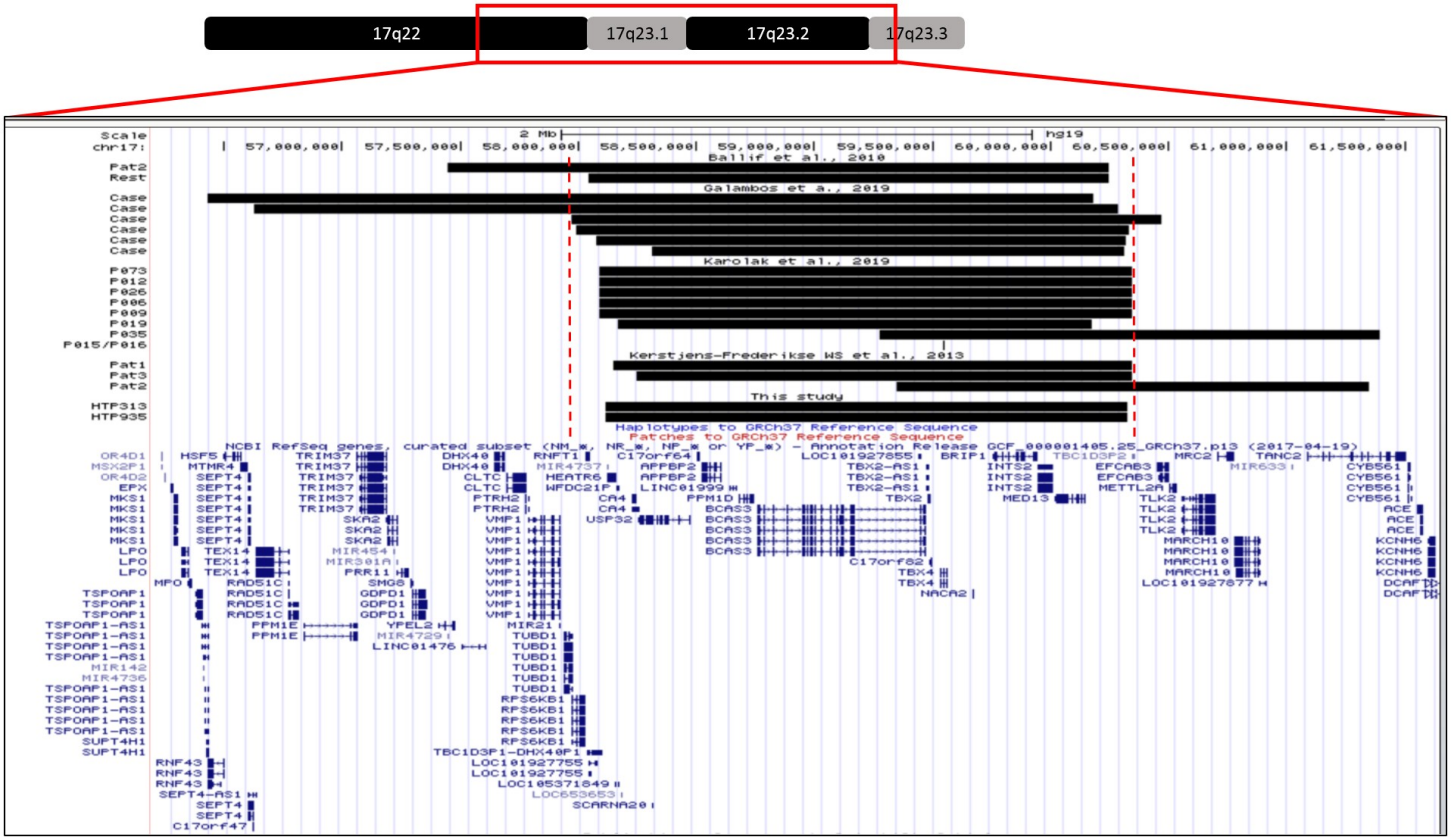
Comparison of different copy number variants reported.

In our study, we have also detected two de novo deletions of one copy of TBX4 in two unrelated individuals with a pediatric form of PAH. Strikingly, these two deletions exactly the same size (2.2Mb) and encompass 17 genes and potential genes: CA4, LOC645638, HEATR6, LOC653653, SCARNA20, USP32, C17orf64, APPBP2, PPM1D, TBX2, BCAS3, C17orf82, TBX4, NACA2, BRIP1, INTS2 and MED13, suggesting a hotspot region susceptible for rearrangements. Comparing these variants with previously CNVs reported, we observe that many of them have a similar size and involve the same region of chromosome (Fig 18) [7–9,13]. However, in some cases, CNV are larger and involve other genes. These findings might explain different phenotypes observed in these patients, and support the idea of a hot spot region due to a flanking LCR region. 17q23 deletions have previously been associated with lung developmental disorders and neonatal/pediatric PAH [13]. One of our pediatric patients (patient 8) showed features compatible with ILD in imaging studies, needing oxygen in her first year of life, with subsequent improvement. Furthermore, patient 7’s mother had previously had two spontaneous abortions, one of which had pulmonary hypoplasia. We speculate that the fetus also carried this CNV inherited from a parent. These findings are in line with a previous study by Karolak et al. In their studies, CNV deletions do not co-segregate with lung disorders. A loss-of-function variant per se cannot predict a possible phenotype and additional genetic modifiers were required to cause severe lung disease. They provide evidence of the complex compound inheritance of lung maldevelopment consisting of rare coding variants in TBX4 or FGF10 and rare or common non-coding SNVs. Further investigation is necessary to address the role of genetic and non-genetic modifiers in the phenotype of *TBX4* variants.

## 5. Conclusions

PAH associated with *TBX4* variants shows a wide spectrum of clinical presentations. A severely reduced DLCO is a common finding. Genetic testing is an extremely useful tool for cases with overlapping clinical features, and might even rule out other forms of PH such as PVOD or PAH associated with HHT. In this respect, a PAH-specific gene diagnostic panel is a cost and time-efficient technique to reach an accurate diagnosis.

## Supporting information

S1 File (DOCX)
