# Supplementary material for: Clinical heterogeneity of Pulmonary Arterial Hypertension associated with variants in TBX4
Source: PLoS One. 2020 Apr 29;15(4):e0232216. doi: 10.1371/journal.pone.0232216 (PMC7190146; doi:10.1371/journal.pone.0232216)
Supplement: S1 File — (DOCX) [file pone.0232216.s001.docx]

**Registry of Pediatric Pulmonary Hypertension Centers:**

- **Hospital Universitario de la Paz** - Madrid (Madrid)
  - C. Labrandero
- **Hospital Universitario 12 de octubre** - Madrid (Madrid)
  - A. Mendoza
- **Hospital Universitario Ramón y Cajal** - Madrid (Madrid)
  - M. del Cerro
- **Hospital Universitario Virgen del Rocío** - Sevilla (Sevilla)
  - I. Guillén
- **Complejo Asistencial Universitario de Salamanca** - Salamanca (Salamanca)
  - B. Plata
- **Unidad de Cardiopatías Congénitas (UCC)** - Madrid (Madrid)
  - S. Villagrá
- **Hospital Universitario Vall d´Hebron** - Barcelona (Barcelona)
  - A. Moreno
  - A. Sabaté
- **Hospital General de Segovia** - Segovia (Segovia)
  - S. Jiménez
- **Hospital Universitario Marqués de Valdecilla** - Santander (Cantabria)
  - M. Viadero
- **Hospital Universitario Miguel Servet** - Zaragoza (Zaragoza)
  - M. López
- **Hospital Universitario y Politécnico La Fe** - Valencia (Valencia)
  - A. Moya
- **Hospital Universitario Puerta de Hierro Majadahonda** - Majadahonda (Madrid)
  - A. Siles
- **Hospital Clínico Universitario Virgen de la Arrixaca** - El Palmar (Murcia)
  - J. Espín
- **Hospital Virgen de la Salud** - Toledo (Toledo)
  - G. Íñigo
- **Hospital Regional Universitario de Málaga** - Málaga (Málaga)
  - L. Conejo
- **Hospital Teresa Herrera** - A Coruña (A Coruña)
  - M. Lozano
- **Hospital Universitario Gregorio Marañón** - Madrid (Madrid)
  - A. Rodríguez
- **Hospital Universitario Reina Sofía** - Córdoba (Córdoba)
  - E. Gómez
- **Hospital Universitario Son Espases**- Palma de Mallorca (Islas Baleares)
  - S. Escribá
- **Complejo Hospitalario Universitario Insular Materno-Infantil** - Las Palmas de Gran Canaria (Las Palmas)
  - H. Falcón
- **Hospital Universitario Virgen de las Nieves** - Granada (Granada)
  - F. Perin
- **Hospital Universitario Donostia** - Donostia/San Sebastián (Guipúzcoa)
  - M. Izquierdo
- **Fundación Neumológica Colombiana** - Bogotá (Bogotá)
  - Ó. Barón
- **Clínica Shaio** - Bogotá (Bogotá)
  - L. Caicedo
- **Clínica Cardio VID** - Medellín (Medellín)
  - M. Zapata
- **Fundación Valle del Lili** - Cali (Cali )
  - W. Mosquera
- **Hospital Universitario Río Hortega** - Valladolid (Valladolid)
  - F. Centeno

**Spanish Registry of Pulmonary Arterial Hypertension Centers**

- **Hospital Universitario 12 de Octubre** - Madrid (Madrid)
  - P. Escribano
- **Hospital Universitario y Politécnico La Fe** - Valencia (Valencia)
  - R. López
  - J. Rueda
- **Hospital Universitario Insular de Gran Canaria** - Las Palmas de Gran Canaria (Las Palmas)
  - F. Guerra
- **Hospital Universitario Central de Asturias** - Oviedo (Asturias)
  - P. Bedate
- **Hospital Universitario Marqués de Valdecilla** - Santander (Cantabria)
  - A. Martínez
- **Hospital Universitario A Coruña** - A Coruña (A Coruña)
  - I. Otero
- **Hospital General Universitario de Valencia** - Valencia (Valencia)
  - G. Juan *)*
- **Hospital Universitario Vall d´Hebrón** - Barcelona (Barcelona)
  - A. Román
- **Hospital Universitario Virgen del Rocío** - Sevilla (Sevilla)
  - F. García
  - T. Elías
- **Hospital Universitario Puerta de Hierro - Majadahonda** - Majadahonda (Madrid)
  - J. Segovia
- **Hospital Universitario Miguel Servet** - Zaragoza (Zaragoza)
  - J. Domingo
  - M. López
- **Hospital Virgen de la Salud** - Toledo (Toledo)
  - M. Lázaro
- **Hospital Universitario Son Espases** - Palma de Mallorca (Islas Baleares)
  - E. Sala
- **Hospital Universitario Clínic de Barcelona** - Barcelona (Barcelona)
  - J. Barbera
- **Hospital Universitario La Paz** - Madrid (Madrid)
  - S. Alcolea
- **Hospital El Bierzo** - Ponferrada (León)
  - J. Ortiz
- **Hospital Universitario Basurto** - Bilbao (Bilbao)
  - F. Mazo
- **Hospital General Universitario Los Arcos del Mar Menor** - San Javier (Murcia)
  - F. Martínez
- **Hospitales Universitarios Vall d'Hebron - Sant Pau** - Barcelona (Barcelona)
  - L. Dos
- **Hospital Universitario de Canarias** - San Cristobal de la Laguna (Santa Cruz de Tenerife)
  - A. Lara
- **Hospital General Universitario de Alicante** - Alicante (Alicante)
  - J. Gil
- **Hospital del Mar** - Barcelona (Barcelona)
  - L. Molina
- **Hospital Universitario Ramón y Cajal** - Madrid (Madrid)
  - E. Garrido-Lestache
  - S. del Prado
- **Clínica Universidad de Navarra (Sede Pamplona)** - Pamplona (Navarra)
  - A. Campo
- **Complejo Hospitalario de Navarra** - Pamplona (Navarra)
  - G. Lacuey
- **Hospital Universitario Infanta Leonor** - Madrid (Madrid)
  - V. Suberviola
- **Hospital Jerez de la Frontera** - Jerez de la Frontera (Cádiz)
  - J. Jiménez
- **Hospital Universitario de Gran Canaria Dr. Negrín** - Las Palmas de Gran Canaria (Las Palmas)
  - G. Pérez
- **Hospital Universitario Gregorio Marañón** - Madrid (Madrid)
  - T. Mombiela
- **Hospital Universitario de Salamanca (Clínico)** - Salamanca (Salamanca)
  - S. Cadenas
- **Hospital Clínico Universitario de Valencia** - Valencia (Valencia)
  - M. Marín
- **Hospital Universitario Dr. Peset** - Valencia (Valencia)
  - I. Furest
- **Complejo Hospitalario Universitario Insular-Materno Infantil** - Las Palmas de Gran Canaria
  - E. Martínez-Quintana *(Cardiología)*
- **Hospital Clínico Universitario Virgen de la Arrixaca** - El Palmar (Murcia )
  - F. Pastor
- **Hospital Universitario Rey Juan Carlos** - Móstoles (Madrid)
  - E. Barrios
- **Hospital Universitario Fundación Jiménez Díaz** - Madrid (Madrid)
  - I. Hernández
